# Novel and Engineered Type II CRISPR Systems from Uncultivated Microbes with Broad Genome Editing Capability

**DOI:** 10.1089/crispr.2022.0090

**Published:** 2023-06-01

**Authors:** Lisa M. Alexander, Daniela S. Aliaga Goltsman, Jason Liu, Jyun-Liang Lin, Morayma M. Temoche-Diaz, Sarah M. Laperriere, Andreas Neerincx, Christien Bednarski, Philipp Knyphausen, Andre Cohnen, Justine Albers, Liliana Gonzalez-Osorio, Rodrigo Fregoso Ocampo, Jennifer Oki, Audra E. Devoto, Cindy J. Castelle, Rebecca C. Lamothe, Gregory J. Cost, Cristina N. Butterfield, Brian C. Thomas, Christopher T. Brown

**Affiliations:** ^1^ Metagenomi, Inc., Discovery, Emeryville, California, USA.; ^2^ Bayer AG, Research & Development, Pharmaceuticals, Leverkusen, Germany.; ^3^ Metagenomi Inc., Pre-clinical, Emeryville, California, USA.

## Abstract

Type II Clustered Regularly Interspaced Short Palindromic Repeats (CRISPR)-Cas9 nucleases have been extensively used in biotechnology and therapeutics. However, many applications are not possible owing to the size, targetability, and potential off-target effects associated with currently known systems. In this study, we identified thousands of CRISPR type II effectors by mining an extensive, genome-resolved metagenomics database encompassing hundreds of thousands of microbial genomes. We developed a high-throughput pipeline that enabled us to predict tracrRNA sequences, to design single guide RNAs, and to demonstrate nuclease activity *in vitro* for 41 newly described subgroups. Active systems represent an extensive diversity of protein sequences and guide RNA structures and require diverse protospacer adjacent motifs (PAMs) that collectively expand the known targeting capability of current systems. Several nucleases showed activity levels comparable to or significantly higher than SpCas9, despite being smaller in size. In addition, top systems exhibited low levels of off-target editing in mammalian cells, and PAM-interacting domain engineered chimeras further expanded their targetability. These newly discovered nucleases are attractive enzymes for translation into many applications, including therapeutics.

## Introduction

The discovery of Clustered Regularly Interspaced Short Palindromic Repeats (CRISPR) systems and their repurposing as programmable gene editing tools has enabled significant advances in therapeutics and biotechnology. The core function of CRISPR enzymes is RNA-targeted DNA or RNA interference. For type II systems, such as Cas9, this is accomplished via a genomically encoded tracrRNA that hybridizes with a processed crRNA derived from the eponymous CRISPR array.^1–3^ The CRISPR array consists of a variable spacer region, which specifies the DNA sequence targeted for cleavage and a constant repeat region with complementarity to the tracrRNA. For gene editing applications, these two separate RNA components can be engineered into a single guide RNA (sgRNA).^1^ Although the genomic target sequence is defined by complementarity to the spacer of the crRNA or sgRNA sequence, cleavage does not occur without an adjacent sequence specific to each type II system, known as the protospacer adjacent motif (PAM).

Various type II CRISPR systems have been described from different bacterial organisms, for example, *Streptococcus pyogenes* (SpCas9), *Staphylococcus aureus* (SaCas9), *Campylobacter jejuni* (CjCas9), and *Neisseria meningitidis* (NmCas9).^1,4–6^ Recently, small orthologs from SaCas9 that combine high activity with a comparably permissible PAM have been reported.^7^ Although additional orthologs were mined from public genomic databases based on sequence homology to known Cas9s,^8^ the most widely used system is still SpCas9 due to its well-characterized biochemistry and short PAM (nGG).^1^ Additionally, any system for therapeutic use needs to be rigorously tested for off-targets to establish its safety profile,^9^ and more precise systems are desirable.

Since initial experiments demonstrating the use of type II enzymes for knock outs, there has also been considerable development of CRISPR systems for more targeted genetic changes, for example, via knock-in homology-directed repair (HDR) or base editing.^10^ The set of previously identified PAMs currently limits any approach that requires precise genomic targeting.^11^ As such, there is a critical need for identifying type II enzymes with novel PAMs and small sizes. We mined metagenomics databases for novel type II systems, which we then tested *in vitro*, in *Escherichia coli*, and in mammalian cells for cleavage activity and specificity, resulting in a diverse set of enzymes with improved activities and diverse PAM compatibilities.

The modular nature of Cas9 effectors^12,13^ has enabled engineering novel PAM compatibility. For instance, PAM specificity of Nme1Cas9 can be altered by swapping its PAM interaction domain (PID) with close orthologs.^14^ However, it has not been shown whether the PID can be recombined from distantly related orthologs. We engineered one of the most efficient nucleases identified in this study, MG3-6, to demonstrate PAM swapping capabilities to alter its targeting specificity. This engineering approach can be employed for proteins both from within and between distantly related subgroups.

## Materials and Methods

### Type II nuclease identification

Publicly available metagenomic sequencing data were downloaded from the public repository NCBI sequence read archive (SRA). In addition, 19 animal microbiome, high temperature biofilm, and sediment samples were collected and stored on ice or in Zymo DNA/RNA Shield after collection. DNA was extracted from samples using either the Qiagen DNeasy PowerSoil Kit or the ZymoBIOMICS DNA Miniprep Kit. DNA sequencing libraries were constructed (Illumina TruSeq) and sequenced on an Illumina HiSeq 4000 or NovaSeq at the Vincent J. Coates Genomics Sequencing Laboratory at UC Berkeley, with paired 150 base pair (bp) reads with a 400–800 bp target insert size (10 gigabases [∼60 M reads] of sequencing was targeted per sample). Metagenomic data are available in the SRA under BioProject accession number PRJNA874471. Sequencing reads were trimmed using BBMap (sourceforge.net/projects/bbmap)^15^ and assembled with Megahit v12.^16^ Open reading frames (ORFs) and protein sequences were predicted with Prodigal v13.^17^

Hidden Markov Models (HMMs) profiles of known type II CRISPR nucleases were searched against all predicted proteins using HMMER3 (hmmer.org) to identify homologs of type II CRISPR-associated (Cas) proteins; HMMs were obtained from Burstein et al.^18^ and the TIGRFAM database.^19^ These hits were aligned with the software MAFFT (default parameters) and used to make new HMMs with HMMER3. Catalytic residue annotations were predicted from a multiple sequence alignment with the reference SaCas9 protein sequence where residues D10, E477, and D702 are from RuvC domains, and H557, N580, and H701 are from the HNH domain. CRISPR arrays were predicted on assembled contigs with Minced (https://github.com/ctSkennerton/minced).^20^ For most Type II nucleases nominated for characterization in the laboratory, we confirmed that the CRISPR locus was accurately assembled by Megahit by visualizing the reads mapping back to the contig. In all cases, the sequence reads support the correct assembly of the locus.

### Phylogenetic analysis

**FIG. 1.**
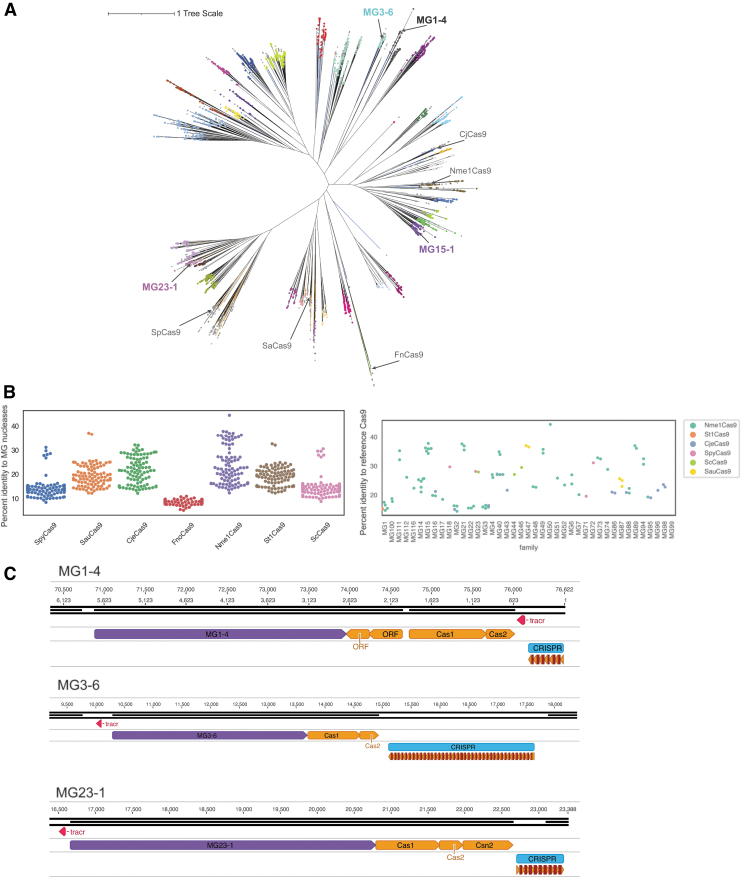
Bioinformatic identification of type II CRISPR effectors. **(A)** Tree of type II CRISPR effectors. Reference sequences are annotated with colored lines according to their reported classification where orange = II-A, green = II-B, and blue = II-C. Reference sequence lines terminate with a gray dot. Subgroups of new systems described in this article are shown with colored dots. **(B)** Percent identity distribution of active nucleases reported here versus reference Cas9 sequences. Left panel: Percent identity of each of the 93 active nucleases described here versus common Cas9 reference sequences. Right panel: Maximum percent identity of 93 active nucleases, ordered by subgroup, to one of eight common Cas9 references, color coded by the reference. **(C)** Genomic contexts of three of our active systems. The nuclease is shown in purple with the tracrRNA in pink and the array is in blue (repeats in orange and spacers in red). CRISPR, Clustered Regularly Interspaced Short Palindromic Repeats; ORF, open reading frame.

We globally aligned all type II homologs identified with MAFFT v7.487 (mafft—large—globalpair)^21^ and built a phylogenetic tree using FastTree^22^ with default parameters. Subgroups were visually identified by tree structure, as well as by the presence of previously characterized reference sequences.^8^ To evaluate the diversity of the identified nucleases, we built a phylogenetic tree with 4483 putative type II effectors identified here and 801 reference sequences (Supplementary Table S1).^8^ The tree was classified into 41 subgroups based on clades in the tree structure (Fig. 1A). Briefly, subgroups within clades on the phylogenetic tree were initially delineated based on whether sequences in that group hit with <50% identity to a reference sequence (Supplementary Table S1). Thus, each subgroup varies in the amount of diversity within. Further analysis of the clades is discussed below and is shown in Supplementary Figure S2. The unmasked alignment, and original and masked trees in Newick format are included as Supplementary Data.

Percent identity distribution plots in Figure 1 and Supplementary Figure S1 were determined from an identity matrix calculated with pseqsid (https://github.com/amaurypm/pseqsid) from the multiple sequence alignment used to infer the phylogenetic tree in Figure 1A.

### Cluster analysis

Cas9 homologs were used to construct a sequence similarity network (SSN). Sequences were dereplicated using MMseq2 (easy-cluster—cov-mode 1—min-seq-id 0.99-c 0.8),^23^ and pairs of homologous sequences were identified by running an all-versus-all Basic Local Alignment Search Tool (BLAST) using DIAMOND blastp (-k0—ultra-sensitive).^24^ The results of an all-versus-all BLAST were filtered to include pairs with percent identities >40% average amino acid identity (AAI) over 50% of the query sequence. The graph was visualized in R using ggraph and clustered using the cluster_fast_greedy function in igraph (Supplementary Fig. S1). Additionally, the original phylogenetic tree was clustered using TreeCluster^25^ using max_clade = 2.5. The two clustering methods were compared visually with the assigned MG subgroups by coloring the above phylogenetic tree according to each clustering method (Supplementary Fig. S2) and additionally compared with each other and with the original MG subgroup designations by calculating the adjusted Rand index, a measure of the similarity between two data clusterings.^26^

### TracrRNA identification

TracrRNAs were predicted as described previously,^27^ with slight modifications. Briefly, we searched for potential anti-repeats matching to the CRISPR repeat along the length of the contig with BLASTN.^28^ The three best anti-repeat hits were considered, based on location (not CRISPR array self hits and within 8000 bp from the CRISPR array) and bit-score. TracrRNAs were extended in the 3′ direction from the anti-repeat hits using information about tracrRNA secondary structure stability^27^ and the presence of a 3′ terminator sequence (polyT sequences ≥4). High confidence tracrRNAs based on minimum folding energy, length, and quality of the anti-repeat were visually examined to confirm boundaries and folding pattern.

Anti-repeats within ORFs were discarded. In some cases, we were able to predict the direction of the CRISPR array transcription due to conserved 5′ motifs such as GTT or GTC. However, we did not limit our search to these cases, and in some contigs, tracrRNAs were predicted in both directions to increase the chances of finding an active guide. RNAfold in the ViennaRNA package was used to calculate the minimum free energy secondary structures for all predicted TracrRNAs.^29^ The RNApdist function was then used to calculate all pairwise distances between thermodynamic secondary structures. The pairwise similarities were visualized using heat map in the R stats package.

### Multiple sequence alignment and structural prediction

Protein sequences were aligned in Geneious Prime using MUSCLE (https://www.geneious.com/features/sequence-alignment). The structure of MG3-6 was predicted by NovaFold and displayed by Protean 3D (NovaFold^®^, Versions 16 and 17; DNASTAR, Madison, WI).

### PID clustering

To extract the PID of all the type II candidates, we first performed an MAFFT global protein alignment with the parameters described above. *S. aureus* (SaCas9) was chosen as the reference type II protein for its well-characterized structure and similar size to the tested effectors. The full-length protein global alignment was trimmed to extract both the wedge (WED) and PIDs found on the C-terminus of SaCas9, starting at amino acid residues 788 and 910, respectively.^30^ To identify the PID boundaries, the sequences from the trimmed alignment were globally aligned again with MAFFT (global alignment), from which we extracted the region corresponding to only the PID, based on the domain boundaries of SaCas9.

This iterative approach to determining the PID boundaries was necessary since the PID sequences may be too divergent, so that the PID alignment was not influenced by more conserved regions from the full-length alignment. PIDs from type II candidates with confirmed PAM sequences were used to construct an SSN. The SSN was constructed as described above, with the exception that the results of the all-versus-all BLAST were filtered to include pairs with percent identities >25% AAI over 50% of the query sequence. The graph was visualized in R using ggraph with the nodes colored according to the PAM sequence hierarchical cluster (see the PAM hierarchical clustering Methods section below).

### Construct design

Nucleases were codon optimized for *E. coli* and ordered in expression plasmids from Twist Biosciences with an N-terminal His tag, N-terminal maltose binding protein (MBP), tobacco etch virus (TEV) site, one nucleoplasmin nuclear localization signal (NLS) at N term, and one SV40 NLS at C term all under control of an inducible ptac promoter. For mammalian expression, the nucleases were codon optimized for human expression and cloned into an expression vector with an N-terminal nucleoplasmin NLS and C-terminal SV40 NLS, followed by a His tag, T2A linker, and green fluorescent protein (GFP). For chimeric constructs, all genes were codon optimized for *E. coli* expression, synthesized, and cloned into pET21 with additions of ribosome binding sites 5′ of translation initiation codons. The compositions of chimeras are listed in Supplementary Table S6.

### sgRNA design

Putative tracrRNAs were identified via identification of anti-repeats based on complementarity to CRISPR repeat sequences, RNA secondary structure stability assessment, and terminator sequence prediction (described above in section TracrRNA identification). TracrRNAs were then folded with the repeat using Vienna RNAfold,^29^ trimmed, and connected via a tetraloop sequence. The tetraloop GAAA was used preferentially unless this altered the predicted folding, in which case TTCG was used as a tetraloop instead. Folding is shown in Geneious 2021.2.2 at 37° with the algorithm from Andronescu et al. in 2007.^31^ Although initial testing used one sgRNA design, we later expanded our testing to use eight sgRNA: two different sgRNA scaffold designs based on different point of tetraloop addition, two spacers (40% GC content and 67% GC content), and two spacer lengths (20 and 24 nt). Where multiple spacer lengths or scaffolds were active.

In some cases where a tracrRNA cannot be predicted, we can use *E. coli* cell extract to transcribe the unknown tracrRNA from the noncoding (intergenic) sequence and use RNAseq to identify the active tracrRNA. In this case, we use an *E. coli* cell lysate kit (myTXTL T7 Expression Kit; Arbor Biosciences) and add three expression templates at a 5:12:15 nM ratio: the nuclease under control of a T7 promoter, a minimal array of repeat-spacer-repeat under a T7 promoter, and a DNA fragment, which corresponds to intergenic regions of interest from the native contig where a tracrRNA might be found. This fragment is expected to produce a tracrRNA due to transcription from an unknown native promoter.

After expression at 29°C for 16 h, the reaction is used to test for *in vitro* activity the same as for sgRNA testing (see section *In vitro* activity assay below). For RNA sequencing, RNA was extracted from 10 μL of the myTXTL reaction using the Quick RNA mini-prep kit (Zymo) and then prepared for sequencing using a small RNA library prep kit (NEBNext^®^ Small RNA Library Prep or RealSeq-AC from SomaGenics). The reads were mapped back to the templates using BBMap in Geneious.^15^ Following RNAseq, sgRNA were designed and validated using the standard *in vitro* activity workflow.

### *In vitro* activity assay

Screening of the nucleases and chimeras was accomplished via a modification of previously described protocols.^32–34^ The modified *in vitro* screen allows for high-throughput characterization of the PAM sequences without protein purification by using an *in vitro* transcription/translation system and either *in vitro* transcribed or chemically synthesized sgRNA. First, the nucleases are expressed in either an *E. coli* lysate expression system (myTXTL T7 Expression Kit; Arbor Biosciences) or a recombinant system (PURExpress; NEB). In both cases, 5 nM of a polymerase chain reaction (PCR)-generated template is added before expression is conducted according to kit recommendations. Generally, the recombinant system has much higher signal and is preferred. After expression, the nuclease mixture is diluted 5-fold (for TXTL) or 10-fold (for PURExpress) into the digest mixture, containing 5 nM PAM library plasmid and 50 nM sgRNA in 10 mM Tris, pH 7.5, 100 mM NaCl, and 10 mM MgCl_2_.

After 1 h, the digest is cleaned up via solid-phase reversible immobilization (SPRI) beads and eluted in Tris-EDTA (TE). 1.5 nM of digested plasmid is ligated to adapters by blunt-ended ligation with 150 nM double-stranded adapter oligos with T4 ligase (final 20 U/μL) in 1 × T4 ligase buffer (NEB). The ligation product is used as a template for Next-Generation Sequencing (NGS) library preparation for standard amplicon sequencing for 150 bp single-end read with a target depth of 50,000 reads. Reads were filtered by quality score Phred quality score >20. Twenty-eight base pairs from the backbone adjacent to the PAM were used as a reference to find the PAM and extracted for alignment. The distance between the PAM and the ligated adapter was also measured for each read to determine the cut site. Reads that did not have an exact match to the reference sequence or adapter sequence were excluded. SeqLogos were generated with Logomaker^35^ using reads restricted to ±2 nt of the cut site as determined by the highest observed site of cutting.

PAM sequences were determined by the height of each nucleotide in a SeqLogo,^36^ namely multiplying the maximum uncertainty, bits, per position by the frequency of each observed nucleotide, and all bases above a threshold of 0.1 were included in the final PAM sequence. PAM sequence logos are shown in Supplementary Figure S6, and the called PAMs are shown in Supplementary Table S2. Given all possible variants of an 8 nucleotide string, we calculate that the 89 unique PAMs described in this article are capable of targeting 59,392 sequences, or 90% of the 8N sequence space. To calculate this, we enumerated all possible 8N PAMs and then converted each Type II PAM into a regex string, where for example PAM NANNHYNN became “[ATGC][A][ATGC][ATGC][ACT][CT][ATGC][ATGC]” and identified cumulative matches between each type II regex'd PAM and all 8N PAMs.

### PAM hierarchical clustering

For each active effector, we vectorized the percent frequency values of each nucleotide at all eight PAM positions determined from the *in vitro* PAM assay. We used the vector of percent nucleotide frequencies to calculate the Euclidean distance between all PAMs and generated a pairwise distance matrix for hierarchical clustering. To get distinct groupings by agglomerative hierarchical clustering, we used the clustering package from SciPy with the unweighted pair group and arithmetic mean linkage methods, which was visualized using the dendrogram plotting function.^37^

### *E. coli* activity assay

Mutant BL21 cell lines were generated by co-transforming a dual T7 expression plasmid containing SpCas9 nuclease with a LacZ single guide targeting the sequence GAGGCTGAAGTTCAGATGTG, with a repair plasmid, which contained the engineered spacer, the appropriate PAM and 500 bp of homology to the flanking region of the cut site for HDR. Once clones were verified for genomic editing by colony PCR and sequencing, engineered strains were then cured of all plasmids. For testing activity in *E. coli*, the engineered strains of BL21 were transformed with a plasmid encoding the nuclease of interest under a T7 promoter and grown on Luria Broth (LB)/ampicillin plates overnight.

Colonies from the plate were used to make competent cells according to kit instructions (Zymo Mix and Go) and transformed with 50 ng of targeting or nontargeting sgRNA plasmid. After 2-h recovery, the cells were serially diluted and plated on LB/ampicillin/chloramphenicol/isopropyl β-D-1-thiogalactopyranoside (IPTG) plates. The fold reduction in growth was measured by counting colonies at the dilution factor with 5–30 colonies and back-calculating. For cases where zero colonies were detected (complete repression of growth), an arbitrary colony count of 1 was used to give a lower bound on repression activity and allow for normalization.

### Gene editing activity in HEK293T cells

For measuring activity in mammalian cells, HEK293T cells are seeded at a density of 15,000 cells per well (96-well plate) 1 day before transfection. Cells are transfected with 140 ng effector plasmid and 60 ng guide RNA plasmid per well. After 72 h, cells are trypsinized using TrypLE, and 100 μL phosphate buffered saline (PBS) is added to each well. Ten to 20 μL are transferred to a new plate for crude DNA extraction (using Dilution Buffer and DNA Release Additive from Thermo Fisher). One microliter of crude DNA is used in a barcoded Amplicon PCR for NGS preparation using the 300 bp single end kit, target depth of 20,000 reads. Reads are analyzed for InDel formation using CRISPResso.^38^ For ribonucleoprotein (RNP) testing, a similar procedure was performed, nucleofecting 104 pmol of protein and 120 pmol sgRNA per well.

### Protein purification

For the purification of MG3-6, 3-7, 3-8, 1-4, 7-1, 14-1, autoinducing media was inoculated with fresh plate scraped transformants in BL21 (DE3), and the cultures were incubated at 37° for 3 h and then cooled to 18° and shaken overnight. After the first night, the media was supplemented with IPTG (1 mM) and grown for 48 more hours. After cell harvest, cells were resuspended in lysis buffer (50 mM Na_2_HPO_4_, pH 8, 800 mM NaCl, 10 mM imidazole, BugBuster, Benzonase) and lysed via sonication. The lysate was purified on a HisTrap HP column eluting with a linear gradient from 100 to 500 mM imidazole in 50 mM Na_2_HPO_4_, pH 8, and 100 mM NaCl. Fractions were pooled and diluted to 10 mL, then supplemented with 0.4 mg TEV protease, 1 mM dithiothreitol (DTT), and 1 mM ethylenediaminetetraacetic acid (EDTA), and incubated for 48 h at 4°.

The TEV cleaved product was loaded onto HiTrap SP HP for ion exchange and eluted with a 100 to 1000 mM KCl gradient with 20 mM 4-(2-hydroxyethyl)-1-piperazineethanesulfonic acid (HEPES), pH 7. Relevant fractions were pooled and buffer exchanged using PD-10 columns according to the manufacturer's instructions into storage buffer 40 mM HEPES, pH 7.0, 400 mM KCl. After addition of 80% glycerol and DTT, the storage conditions are 20 mM HEPES, pH 7.0, 200 mM KCl, 1 mM DTT, and 40% glycerol, pH 7.0. Proteins were checked for activity using the fluorescence polarization assay as described in the study by Schmidt et al.^7^

### Off-target analysis in HEK293T cells

To assay off-target sites, HEK293T cells were nucleofected with precomplexed RNP consisting of 104 pmol of the indicated protein and 120 pmol sgRNA using the Lonza 4D electroporation system. In parallel, cells were cotransfected with 5 pmol annealed double stranded oligodeoxynucleotide (dsODN) as described.^39^ After 72 h, cells were trypsinized and genomic DNA (gDNA) extracted using the PureLink gDNA extraction kit and quantified. Four hundred nanograms of high-molecular-weight gDNA was fragmented, end-repaired, and ligated using the NEB FS DNA Library Prep Kit. Fragments between 350 and 600 bp were amplified to enrich for dsODN-proximal regions using dsODN-specific primers in both the positive and negative orientations. Resulting libraries were amplified for NGS on Illumina MiSeq and sequenced as 2 × 150 paired end reads. Reads were analyzed using a modified *guideseq* software package (adapted from http://github.com/aryeelab/guideseq). The Venn diagrams for Supplementary Figure S12 were visualized with BioVenn.^40^

### *In vitro* specificity assay

The *in vitro* specificity test is modified from Pattanayak et al.^41^ to use a circular substrate rather than a concatemeric substrate. The substrate is generated by ligation of a 400 bp PCR product containing the PAM to a library of inserts. The insert library is generated from an oligo, similar to that in the study by Pattanayak et al.^41^ The oligo design has a *Bsa*I site, an internal barcode, an *Nru*I site, a 24 nt spacer of mixed bases at 79:7:7:7 where 79% is the percentage of the on-target base, and then, a *Bcc*I site. The *Bcc*I site has a single nt overhang, which allows the PAM to be swapped without changing the library insert. This 60 nt oligo is made double stranded by annealing to a 17 nt primer and fill-in with Klenow exo- (NEB) for 1 h at 37°, followed by heat inactivation at 75° for 20′ and then *Bcc*I treatment at 37° for 1 h.

The *Bcc*I-digested dsDNA insert is recovered using DNA Clean and Concentrate column (DCC-5, Zymo) clean up columns (Zymo). The “backbone” is PCR amplified from pET41(+) plasmid with *Bsa*I and *Bcc*I sites in the primers, cleaned up via spin column, and digested with *Bcc*I (NEB) before a second cleanup step. The two ligation steps were performed sequentially to maximize the amount of 1 nt ligation product without getting concatemers. First, the *Bcc*I-treated insert and backbone were ligated using TA ligase (NEB) at a 6:1 ratio of insert:backbone at 16° overnight. SPRI beads were used to clean up the ligation and remove excess insert. This product was treated with *Bsa*I-HF v2 (NEB) at 37° for 1 h and then cleaned up via SPRI beads. The final ligation step was performed with T4 ligase at 16° for 16 h with a DNA concentration of 5 nM to minimize intermolecular ligation. Linear DNA is removed via treatment with T5 exonuclease (NEB), and then, the final circular product is cleaned up via spin column (Zymo).

For *in vitro* specificity testing, 4 nM of circular substrate is treated with 40 nM RNP in 10 mM Tris, pH 7.5, 10 mM MgCl_2_, and 100 mM NaCl for 1 h at 37°. The reaction is quenched via cleanup with SPRI beads. 1.5 nM of digested plasmid is ligated to adapters by blunt-ended ligation with 150 nM double-stranded adapter oligos with T4 ligase (final 20 U/μL) in 1 × T4 ligase buffer (NEB). The ligation product is used as a template for NGS library preparation for standard amplicon paired-end sequencing. The paired-end NGS reads are reconstructed to regenerate the spacer on both sides of the cut site. Analysis was performed as described in the study by Pattanayak et al.^41^ to generate enrichment scores accounting for the starting library base distribution.

### Messenger RNA synthesis

The coding sequence for MG3-6_3-4 and SpCas9 was cloned into a pUC19 plasmid, including a RNA-pol T7 promoter along with 5′ and 3′ untranslated regions (UTRs), and a 100 nt polyA tail. One hundred micrograms of plasmid was digested with *Sap*I to linearize it downstream of the polyA tail. The plasmid was subsequently purified with phenol–chloroform and precipitated with 70% ethanol. The DNA pellet was resuspended in 20 μL of nuclease-free water. For *in vitro* transcription, 1 μg of linearized plasmid DNA was added to a 20 μL reaction containing 1 × reaction buffer (40 mM Tris-HCl, pH 7.5, 16.5 mM MgCl2, 50 mM NaCl, 2.5 mM Spermidine, 1 mM DTT) and 750 U of Hi-T7 RNA Polymerase (NEB). The reaction was incubated at 50°C for 1 h. Recently transcribed messenger RNA (mRNA) was purified using MEGAclear transcription Clean-up kit (Thermo Fisher) following the manufacturer's instructions.

### Genome editing in Hepa 1-6 cells using mRNA transfection

The day before transfection, Hepa 1-6 cells were seeded at 70,000 cells per well in 24-well plates. The day of transfection 300 ng of mRNA codifying for MG3-6_3-4 or SpCas9 along with 200 ng of chemically synthesized guides (IDT, Alt-R modifications) was complexed in Lipofectamine Messenger Max following the manufacturer's instructions, and the complexes were added to cells. After 48 hours post-transfections, gDNA was isolated using PureLink gDNA extraction kit following the manufacturer's instructions. The site of interest was PCR amplified, and the InDel formation was evaluated using Sanger sequencing, followed by Inference of CRISPR Edits analysis.^42^

## Results

### *In silico* discovery of diverse type II effectors and tracrRNAs

We mined a database of more than 3.8 billion proteins and 8.2 terabases of assembled genomic content from diverse environments (see the Materials and Methods section). Hidden Markov Models were used to identify putative type II effectors distantly related to known nucleases. Through phylogenetic analysis, we identified 4483 putative type II effectors spanning 41 subgroups (Fig. 1A, colored dots represent subgroups). The proteins in each subgroup were labeled by the MG prefix due to their metagenomic origin and a number for their subgroup; members within a subgroup were subnumbered as MGx-y, for example, MG3-6 is member 6 from subgroup 3. Subgroup assignments were supported by two independent methods: clustering the above phylogenetic tree with TreeCluster^25^ and clustering based on an SSN constructed from full protein sequences (Supplementary Fig. S1).

TreeCluster and SSN clustering methods resulted in 74 and 151 clusters, respectively (Supplementary Table S1; Supplementary Fig. S2A, B). Compared with the phylogenetic method, these clustering methods result in more clusters on average because they treat outliers as their own clusters (where they were considered a single cluster by phylogenetic analysis). These methods largely agreed with the delineated MG subgroups, as confirmed by adjusted Rand index calculations (i.e., measures of similarities between data clusterings (Supplementary Fig. S2C).^26^ Specifically, Rand indices of 0.722, 0.724, and 0.853 were observed when comparing the SSN clusters to MG subgroups, the TreeCluster clusters to MG subgroups, and the TreeCluster clusters to SSN clusters, respectively. A value of 1 indicates that the clustering is exactly the same across the methods, and an adjusted value close to zero is random. Therefore, the MG delineations are similar to the TreeCluster and SSN delineations.

Representative members of each subgroup were selected based on the presence of all catalytic residues in the HNH and RuvC domains and CRISPR arrays near the effector (see the Materials and Methods section) and were further tested for *in vitro* nuclease activity. These high-quality effectors range from 978 to 1533 aa in length and are present on contigs from diverse bacterial and viral genomes. Novel nucleases recovered here share at most 50% identity to previously characterized Cas9 nucleases (Fig. 1B, percent identity distribution), including 84 biochemically diverse Cas9 homologs reported recently (Supplementary Fig. S3).^8^

We predicted tracrRNA sequences (see the Materials and Methods section) for all effectors nominated for initial *in vitro* testing. TracrRNA sequences were identified upstream or downstream from the effectors but were most commonly observed in the reverse orientation (69.8% of candidates) and upstream of the effector (79.2% of candidates) (Fig. 1C; Supplementary Table S2). The tracrRNA structures of active candidates were compared, revealing general conservation within subgroups (Supplementary Fig. S4). This observation enabled refinement of tracrRNA prediction for additional members of that subgroup. TracrRNA conservation within subgroups was confirmed from pairwise distance analyses between thermodynamic tracrRNA secondary structures (see the Materials and Methods section) (Supplementary Fig. S5). Many predicted tracrRNAs do not follow the “canonical” folding in their secondary structure prediction [lower stem, bulge, upper stem, nexus, terminal hairpin(s)] observed for SpCas9 or SaCas9 (Supplementary Fig. S4).^43,44^

### *In vitro* testing of nucleases confirmed system activity, sgRNA designs, and identified PAM sequence requirements

**FIG. 2.**
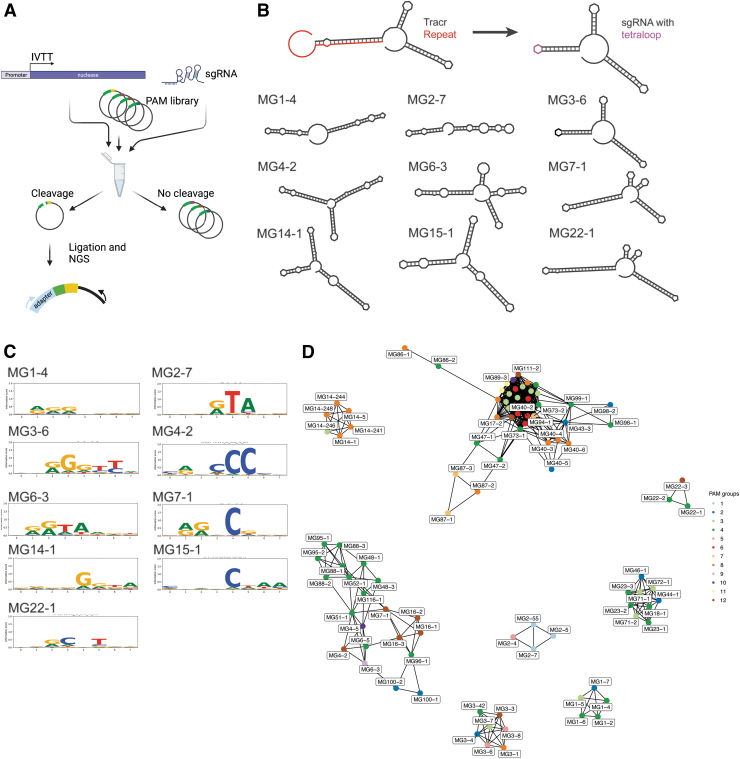
*In vitro* screen of nuclease activity and PAM analysis. **(A)** *In vitro* screen uses *in vitro*-translated protein to target an 8N PAM library. After cleavage, the linear DNA products are sequenced by NGS to recover the PAM. Created with BioRender.com **(B)** sgRNA design is accomplished by truncating the repeat-anti-repeat duplex and joining them together with a tetraloop.^1^ Designs can then be evaluated by visual inspection of the predicted tracrRNA and repeat. Example active sgRNAs from nine different subgroups are shown. **(C)** Example PAMs for the same sgRNA are shown. **(D)** A sequence similarity network of the PIDs of 96 active nucleases colored according to the PAM sequence group. IVTT, *in vitro* transcription-translation; PAM, protospacer adjacent motif; PIDs, PAM-interacting domains; NGS, next-generation sequencing; sgRNA, single guide RNA.

Screening of the newly recovered nucleases for *in vitro* cleavage activity was accomplished via a modification of the protocol described in references^32–34^ (see the Materials and Methods section). We used a high-throughput cell-free PAM enrichment assay to test effector and sgRNA activity and to determine PAM sequence requirements (up to eight sgRNA guide designs for each nuclease were evaluated). After incubation with an 8N 3′ PAM library, the cleaved sequences can be recovered via ligation and subsequent PCR amplification and analyzed via NGS (Fig. 2A).

In total, 96 of 136 tested type II nucleases from 41 diverse subgroups showed cleavage activity *in vitro* (Fig. 2B, C; Supplementary Fig. S6). For nucleases where multiple guide designs were active, the guide that gave the strongest signal was chosen as the optimal sgRNA, or, if equivalent activity, the shorter guide was preferred (Supplementary Fig. S6). For a subset of cases where we could not predict a tracrRNA confidently, we experimentally determined the tracrRNA via expression of the intergenic regions in *E. coli* cell extract relying on endogenous promoter expression and subsequent RNAseq analysis, followed by validation via *in vitro* transcribed sgRNA (Supplementary Fig. S7).

It is noteworthy that we did not perform salt, pH, metal, or temperature optimizations. Therefore, our results represent a lower bound of active proteins in our data sets. The PAMs recovered here encompass a diverse sequence space that may enable a variety of genome editing applications. Collectively, all 96 PAMs described can theoretically target 90% of all possible 8N sequences.

We sought to determine the correlation, if any, between the laboratory-verified PAM sequences and the sequence information in the PID. To make this comparison, an SSN was constructed using 96 PIDs from active candidates (Fig. 2D), and the nucleotide frequencies at each PAM position were used to build a Euclidean distance matrix (Supplementary Fig. S8). Clustering of the SSN resulted in 10 distinct PID clusters, and hierarchical PAM clustering resulted in 12 groups of motifs (Supplementary Figs. S8 and S9). The PID and PAM sequence clusters show little concordance based on the adjusted Rand index of 0.065, suggesting that the PID is not predictive of *in vitro* PAM (Fig. 2D). For example, in PAM group 1 (MG2 subgroup), the PID and PAM cluster together (Fig. 2D; Supplementary Figs. S8 and S9). However, distantly related PID also have similar PAMs as shown for PAM group 6 in Figure 2D (multiple subgroups) (Supplementary Figs. S8 and S9). Additionally, the nucleases have diverse PAMs within and across subgroups, with nucleases within different subgroups sharing similar PAMs.

### *E. coli* and HEK293T experiments identified systems with activity in cells

**FIG. 3.**
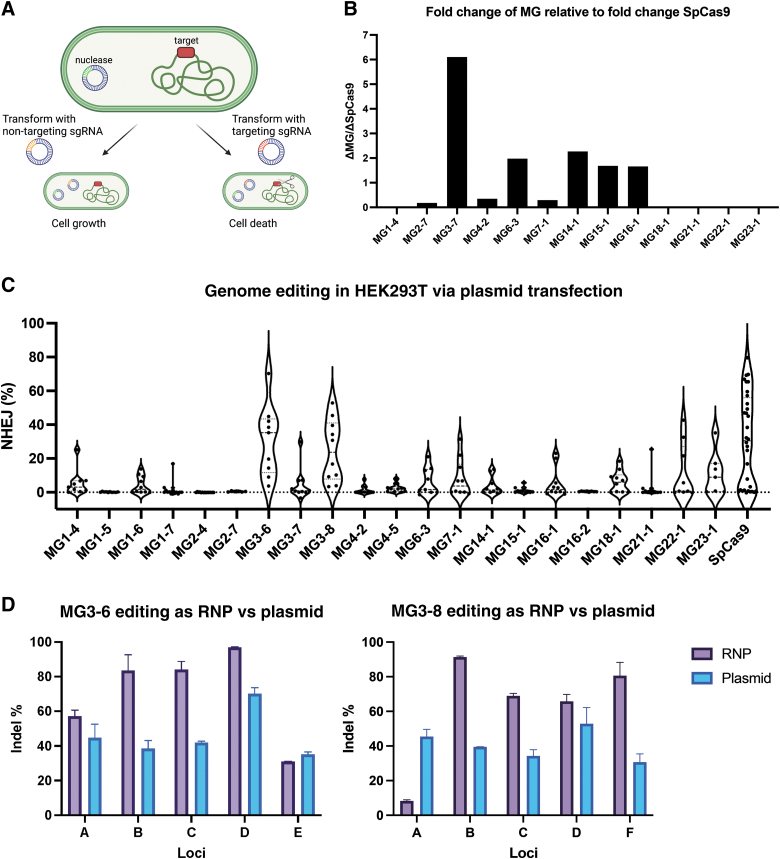
Activity of novel nucleases in *Escherichia coli* and HEK293T cells. **(A)** Design of *E. coli* activity assay. An *E. coli* strain with a mutated genomic target site is transformed with the nuclease and then a targeting or non-targeting sgRNA. Targeting sgRNA will cleave the genome and lead to cell death, measured as fold-repression of growth. Created with BioRender.com **(B)** The activity of the discovered nucleases relative to SpCas9 activity. Each experiment was conducted in triplicate and normalized to same-day SpCas9 controls. **(C)** The activity of the discovered nucleases in HEK293T cells. For each MG nuclease, 8–12 target sites were chosen. Each data point is *N* = 2 for one target site. **(D)** RNP delivery resulted in higher editing on average than plasmid for both MG3-6 and MG3-8 (*N* = 2, error is standard deviation). Four of the target sites are shared between candidates in this screen (sites A–D). RNP, ribonucleoprotein.

Following the proof of *in vitro* activity screen, which validated the functionality of effector sequences, sgRNA sequence, and determined the PAM, we next sought to further characterize our systems in a cellular context in *E. coli* and in mammalian cells. For 13 selected candidates, an *E. coli* strain was developed that incorporated a protospacer with the appropriate PAM sequence into the LacZ gene. After transforming the cells with plasmids containing the enzyme of interest and a targeting sgRNA, we measured enzyme efficiency by the reduction in growth due to genomic cleavage compared with expression of a non-targeting control sgRNA (Fig. 3A). All experiments were conducted in triplicate and also conducted with a simultaneous triplicate transformation of a SpCas9 control.

For each enzyme, we compute the growth repression as the ratio of cfu/μg for the non-targeting sgRNA compared with the targeting sgRNA. This ratio is then compared with the ratio observed for SpCas9, which is included across all experiment days. Nine of 13 candidates are active in *E. coli* (Fig. 3B; Supplementary Fig. S10). This measure is a combination of protein expressibility, guide RNA folding and expressibility, complex formation, cleavage rate, as well as other factors, so poorly performing proteins may be too slow to work well in bacteria or have low overall expression. Five of our candidates show higher growth repression than SpCas9, demonstrating robust overall cleavage and expression by the novel enzymes in *E. coli*.

Although cleavage in bacteria is one possible test of activity, it lacks many of the challenges inherent to gene editing in mammalian cells, for example, the nuclear membrane and chromatin structure. Many CRISPR proteins of interest that show good *in vitro* activity or activity in bacterial systems have difficulty translating into mammalian cells.^3^ To test activity in mammalian cells, we designed expression plasmids for 21 effector enzymes with 2 NLS, one at each termini, as well as 8–12 sgRNA plasmids per effector for 8–12 targeting sites within the human genome. These target sites were across 12 different loci with known targetability by SpCas9. If the PAMs contained degenerate bases, the sites were selected to span a variety of PAM sequences within the *in vitro* determined PAM. The effector and sgRNA plasmid were transfected into Human Embryonic Kidney 293T cells (HEK293T) cells, harvested 72 h later, and analyzed for InDel activity via NGS.

Evidence of InDels at >5% frequency was taken as evidence of active proteins. Using this cutoff, 17 of the 21 proteins were active at at least one target site (Fig. 3C; Supplementary Table S3). All the proteins, including SpCas9, have some targets with <5% editing, indistinguishable from background, showing the limitations of a small guide screen and loci-specific effects for genomic cleavage. Despite having a relatively small guide screen, we were able to show activity of highly novel proteins and sgRNA, for example, three nucleases from the MG1 subgroup, which has the lowest identity of any of our subgroups to known nucleases and very unique tracrRNA structure, were active in cells. We were also able to identify candidates with high relative overall activity across a range of target sites, for example, MG3-6 and MG3-8 and to a lesser extent MG22-1, MG23-1, or MG7-1.

Using the data from the HEK293T screen, we selected six candidates for protein purification and subsequent testing as an RNP complex. We included our two highest activity candidates, MG3-6 and MG3-8, as well as middle and low performing candidates 7-1, 3-7, 14-1, and 1-4 to observe if RNP delivery improved activity. For the top performing candidates MG3-6 and MG3-8 via plasmid activity, RNP delivery boosted the genome editing efficiency to over 90% efficiency at certain target sites (Fig. 3D; Supplementary Table S4). For the other candidates, however, low stability and low activity from protein purification likely led to lower overall editing at the top target sites compared with plasmid transfection (Supplementary Fig. S11).

### MG3 subgroup effectors have high specificity *in vitro* and in mammalian cells

**FIG. 4.**
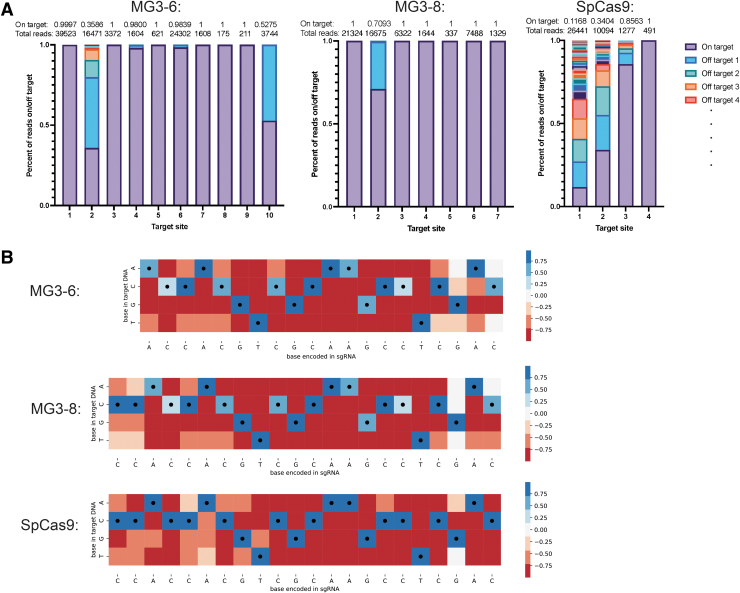
In cell and *in vitro* specificity reveals high fidelity of novel MG3 nucleases. **(A)** Off-target analysis shows that MG3-6 and MG3-8 have high specificity as measured by number of off-target sites and fraction of on-target reads. The target sites are the same between the three candidates. Off-target experiments were conducted at *N* = 2, and reads for both replicates were summed for analysis. **(B)** *In vitro* cleavage data with a library of spacer mismatches show positions where mismatches are most likely to be tolerated. The black dots are the coded bases in the spacer, and the color coding ranges from +1 (100% enriched, dark blue) to −1 (completely de-enriched, dark red). MG3-6 and MG3-8 generally have mismatches tolerated at positions 1–3 with some weaker allowance >16. SpCas9 has stronger preference near the PAM but allows mismatches >16.

Having identified two high-performing candidates for editing via either plasmid or RNP delivery, we sought to characterize these proteins for specificity in mammalian cells. The PAMs of these two systems, nnRRRTY and nnRGRTY for MG3-6 and MG3-8, respectively, enable us to test editing activity at target sites that can also be targeted by SpCas9 (nGG PAM). We selected 10 sites with high editing via RNP delivery for off-target analysis with MG3-6 (9 sites with >90% editing and another with 59% editing), as well as 7 shared target sites for MG3-8 and four shared target sites for SpCas9. These sites were assayed for off-targets through double-strand break discovery via capture of a double-stranded oligonucleotide. For MG3-6, 6 of 10 target sites had <1% off-target reads and 8 of 10 had <2% off-target reads (Fig. 4A; Supplementary Table S5). For MG3-8, 6 of the 7 sites had no detected off-targets.

Analyzing the same target sites (sites 1–4) for MG3-6 and MG3-8 showed that in general they had fewer off-target sites than SpCas9 (Fig. 4A; Supplementary Fig. S12). Target site 1, for example, is a known hot spot for off-targets with SpCas9, where only 11.68% of reads for SpCa9 are on target, with 75 different off-targets, as previously described.^39^ MG3-6 and MG3-8, however, have only two and zero off-target sites, respectively, for this location, suggesting comparable or better specificity than SpCas9. In cases where SpCas9 has zero off-targets, such as target site 4, MG3-6 and MG3-8 have very few or no off-targets as well. Generally, across the seven shared sites between MG3-6 and MG3-8, MG3-8 has fewer off-targets detected. However, both nucleases have very high specificity. The detected off-targets for MG3-6 and SpCas9 are not the same sites in the genome (Supplementary Fig. S12), suggesting some protein-specific mismatch tolerance at different positions in the target.

To assess these position-specific effects, we used purified protein to assay the specificity of the enzyme in a library wide screen *in vitro*. We modified a protocol for a library screen to use a mini-circle substrate.^41^ The mini-circle libraries contain on average five mismatches per spacer and a constant PAM region. This library contains many more possible off-target sites that exist naturally in the genome for a given spacer, allowing us to probe for the full substrate specificity in a less biased way. Across SpCas9, MG3-6, and MG3-8, we observe varied patterns of base preferences, with SpCas9 showing the most mismatches at bases >16 nucleotides away from the PAM (Fig. 4B). MG3-6 and MG3-8, conversely, show fewer mismatches overall and more tolerance at bases 1–3 from the PAM and >20 nt (Fig. 4B). This shows the mismatch profile is not generalizable for type II systems, and further study is warranted to predict which off-targets will be most problematic for a given system and to score potential target sites.

### Altering the PAM requirement through protein engineering enables genome editing at additional targets

Although MG3-6 showed very high editing efficiency and low off-target profiles in mammalian cells, its targetability is limited by its stringent PAM recognition. To expand this capability, we took a protein engineering approach. We included 26 nucleases from diverse protein subgroups and sought to alter the PAM requirement of MG3-6 by domain swapping. Multiple sequence alignment revealed that there were two highly conserved residues (DA at position 1442 from the multiple sequence alignment) located close to C-termini of these enzymes (Supplementary Fig. S13). By taking the structural information from *S. aureus* Cas9, we noticed that a breakpoint at this position could include a portion of RuvC-III, WED, and PAM-interacting (PI) domain if the C-terminal domains were swapped (Supplementary Fig. S13).

**FIG. 5.**
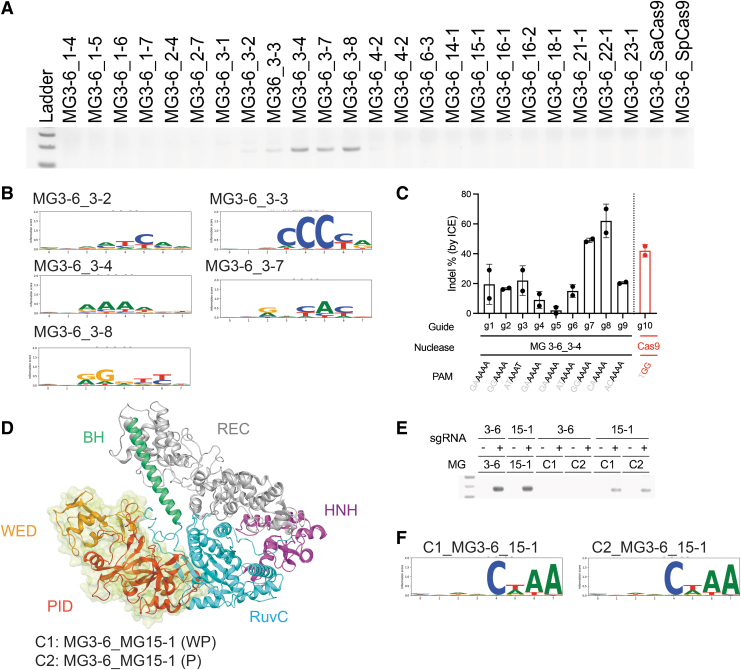
Chimeric proteins are active *in vitro* and in cells. **(A)** Screening of MG3-6 PID swaps shows activity only within the MG3 subgroup. **(B)** Seqlogos for the chimeric proteins for MG3-6_3-2, MG3-6_3-3, MG3-6_3-4, MG3-6_3-7, and MG3-6_3-8. The chimeric PAMs match the PAM of the swapped PID, although sometimes the intensity of the signal can vary. **(C)** The Type II MG3-6_3-4 chimera is highly active in mammalian cells. Editing efficiencies across a variety of guides in Hepa 1-6 cells are shown. SpCas9 with a highly active guide is included as a comparison (g10). *N* = 2, error is standard deviation. **(D)** Different breakpoints for the domains allow swapping across subgroups. The predicted domains of MG3-6 are displayed in different colors. We constructed two chimeras using different breakpoints, C1 and C2. **(E)** Screen data for MG3-6 WT, 15-1 WT, and the C1 and C2 domain swaps with different sgRNA. The first four lanes show the parental activity. Next (lanes 5–8) C1 and C2 are tested with the MG3-6 sgRNA and then with 15-1 sgRNA (lanes 9–12). **(F)** PAMs for MG3-6_15-1 C1 and MG3-6_15-1 C2. WT, Wild type.

As such, we recombined proteins by taking the N-terminal portion from MG3-6 and the C-terminal portion from other enzymes (Supplementary Table S6). We discovered that chimeric proteins could be functional if they were recombined within the same subgroup with this breakpoint (Fig. 5A). In this screening experiment, we used sgRNA from MG3-6 to enable programmable targeting. Failure of recombining proteins across subgroups might result from structural disruption.^45^ We then analyzed the PAM specificities of the functional chimeras and found that they exhibited equivalent or similar recognition as their C-terminal parents (Fig. 5B). For example, the chimera MG3-6_3-4 recognized the MG3-4 PAM nnRAAA, whereas the chimera MG3-6_3-3 recognized the MG3-3 PAM nnnCCCYR, both very distinct from the parental nnRRRTY PAM.

We further showed activity of the MG3-6_3-4 chimera in a mammalian cell context via mRNA transfection in Hepa1-6 cells. This chimera was chosen as our test system because it shifts the PAM from G rich to A rich, which opens up additional target space. For example, genome editing on the intron of albumin locus has been shown to be promising for treatment of hemophilia.^46^ However, the A-rich sequence of albumin restricts applications of MG3-6 targeting. The chimera MG3-6_3-4 we engineered was able to target multiple loci on the intron of mouse albumin gene. Of 20 tested guides, nine showed gene editing activities, and MG3-6_3-4 with guides seven or eight outperformed SpCas9 with its top performing guide, guide 10 (Fig. 5C).

While domain swapping is useful for rapid exchange of PAM compatibility, the utility of a single design can be constrained by sequence homology and structural complexity, as evidenced by the limited hits to our initial chimeric swap design. Therefore, we explored more breakpoint designs to allow the possibility of recombining proteins across subgroups. We predicted the domain architectures of MG3-6 and MG15-1 via 3D modeling (see the Materials and Methods section) and designed two chimeras comprising an N-terminal chassis from MG3-6 linked to a C-terminal section from MG15-1. Proteins were broken at the predicted floppy loops before the wedge domain (chimera C1) or PI domain (chimera C2) (Fig. 5D). Guides from MG3-6 and MG15-1 were tested for nuclease activity. We observed higher activities when the sgRNA from MG15-1 was used, demonstrating the importance of guide selection (Fig. 5E). After NGS analysis of PCR products, our results indicated that both chimeras are active and recognize the PAM CWAA, a PAM that is similar to the native PAM for MG15-1 (Fig. 5f).

## Discussion

Recently, the overall diversity of type II CRISPR enzymes has come under greater scrutiny, and there has been a strong interest in mining type II systems for genome editing applications. Here, we describe 96 novel active type II systems, of which 21 have been screened in mammalian cells. We show how a pipeline of *in vitro* screening through mammalian testing and specificity analysis can funnel a broad starting set of enzymes to identify potent gene editors that are comparable or better than the currently used systems. Our companion article by Lamothe et al. demonstrates the use of MG3-6 and MG3-6_3-4 as powerful gene editors in a variety of therapeutically relevant cell types and contexts, including knock-outs, knock-ins, and for multiplex editing.

One hurdle of translation into mammalian cells is the relatively poor performance of many of the type II systems once in a cellular context so including this in the screening pipeline beyond initial identification and *in vitro* activity is critical. Although most (17 of 21) of our enzymes were active in mammalian cells targeting at least one site, many had low overall efficiency or only showed activity at a small number of sites. The drivers of these site-specific effects are still being investigated. Previously, some systems with low overall cleavage have been demonstrated to have improved cleavage when tested in conjunction with dCas9 to open up nearby chromatin structure,^47^ which may also be beneficial for these new systems.

Also, some of the candidates may be limited in their apparent activity due to more subtle PAM preferences *in vivo* than *in vitro*. This potential effect is explored in our companion article by Lamothe et al., which demonstrates an in cell PAM refinement assay applied to our top performing nucleases, where the PAM for 3–6 was refined from nnRRRTY to nnRGRYY. Despite these factors, the top performing nucleases displayed high efficiency in mammalian cells on a broad range of targets, proving the benefit of taking a wide screening approach.

We also show high specificity in cells for these candidates with unique off-target profiles. In future work, we hope to be able to score potential off-target sites as has been performed for other type II nucleases by combining the *in vitro* specificity data from RNP titrations or time courses along with additional in cell data.

Additionally, we showed how our top performing protein can be engineered into chimeric proteins to expand PAM targeting from a G-rich native PAM to a C or A rich alternative PAM while maintaining high levels of activity. We further show active chimeric constructs between proteins with <20% identity from different subgroups, unlike previous reports where chimeric proteins were 89.2% identical^48^ or >80% identical.^14^ This approach reaps the benefits of metagenomic diversity while limiting the hindrance of low activity when translating many of the natural systems into cells.

The diversity of the described proteins and tracrRNAs is expected to be beneficial for next-generation CRISPR tools that require smaller sizes or unique fusion proteins. The DNA sequence targetability is broad due to the spectrum of nucleases we have discovered as well as the added possibility of recombining protein domains into novel chimeras that we demonstrated. Overall, we anticipate that this expanded collection of richly described type II effector proteins will unlock many applications in biotechnology and medicine.

There are limitations of current systems in gene editing applications, which can also be addressed with this work. Off-target analyses for SpCas9 have also indicated that certain guides may be active at undesirable sites,^49,50^ highlighting the need for more specific systems. Furthermore, recent experiments have demonstrated the limitations of SpCas9, as many individuals have a pre-existing immune response to SpCas9 or SaCas9, which is derived from a human pathogen.^51^ Systems from more diverse sources may have lower immunogenicity in human populations. The companion article by Lamothe et al. demonstrates that nucleases described by our group have a lower immune response and high specificity, allowing us to harness natural diversity to build the next-generation gene editing tools.

## Supplementary Material

Supplemental data
