# Supplementary material for: Novel and Engineered Type II CRISPR Systems from Uncultivated Microbes with Broad Genome Editing Capability
Source: CRISPR J. 2023 Jun 1;6(3):261–77. doi: 10.1089/crispr.2022.0090 (PMC10278012; doi:10.1089/crispr.2022.0090)
Supplement: Supplemental data [file Supp-Data.zip › Alexander_SI_revised_round2.docx]

## Supplemental Figures

## Novel and engineered type II CRISPR systems from uncultivated microbes with broad genome editing capability, Alexander et al.

**Supplementary Data File:** The unmasked alignment and original tree inferred from this alignment for Figure 1, as well as masked trees in Newick format

**Supplementary Table 1: Sequences and reference sequences for Fig. 1A as well as PID boundaries.**

See supplemental excel file.

**Supplementary Table 2: Protein sequences, tracrRNA sequences, sgRNA sequences and contig context information for active nucleases.**

See supplemental excel file

**Supplementary Table 3: Target sites and indel data for HEK293T editing experiments in Fig. 3C**

See supplemental excel file

**Supplementary Table 4: Target sites and indel data for RNP vs plasmid experiments**

|  |  |  |  |  | RNP | | Plasmid | |
| --- | --- | --- | --- | --- | --- | --- | --- | --- |
| Protein | target site | Spacer | PAM | locus | Average indel (N=2) | Standard Deviation | Average indel (N=2) | Standard Deviation |
| MG3-6 | A | TTCTATTTATGAGATCAACAGC | ACAGGTT | Albumin | 57.14 | 3.55 | 44.83 | 7.71 |
| MG3-6 | B | GAGGCCCTGGGCAGGTTGGTAT | CAAGGTT | HBB_R01 | 83.525 | 9.09 | 38.445 | 4.69 |
| MG3-6 | C | AGGTTGGTATCAAGGTTACAAG | ACAGGTT | HBB_R01 | 84.095 | 4.73 | 41.98 | 0.69 |
| MG3-6 | D | TTTAAATTATGAATCCATCTCT | AAAGGTT | Fibrinogen | 96.87 | 0.37 | 70.245 | 3.42 |
| MG3-6 | E | TCAACTTTCCTGGCAACTTGCG | GTAGGTT | ApolipoproteinT524 | 31.055 | 0.16 | 35.24 | 1.27 |
| MG3-8 | A | TTCTATTTATGAGATCAACAGC | ACAGGTT | Albumin | 8.275 | 0.66 | 45.425 | 4.16 |
| MG3-8 | B | AGGTTGGTATCAAGGTTACAAG | CAAGGTT | HBB_R01 | 91.38 | 0.58 | 39.565 | 0.09 |
| MG3-8 | C | AGGTTGGTATCAAGGTTACAAG | ACAGGTT | HBB_R01 | 68.95 | 1.43 | 34.25 | 3.63 |
| MG3-8 | D | TTTAAATTATGAATCCATCTCT | AAAGGTT | Fibrinogen | 65.81 | 3.96 | 52.88 | 9.33 |
| MG3-8 | F | GAAGTTGGTGGTGAGGCCCTGG | GCAGGTT | HBB_R01 | 80.585 | 7.69 | 30.71 | 4.75 |

**Supplementary Table 5: Target sites and summary data for off-target experiments**

|  |  |  |  |  | Summary of dsODN experiment | | |
| --- | --- | --- | --- | --- | --- | --- | --- |
| Protein | Target | Spacer | PAM | Indel %  (N=2) | Total Reads | Fraction on target | Number of off target sites |
| MG3-6 | 1 | CGGTGGCACTGCGGCTGGAGGT | GGGGGTT | 98.5 | 39523 | 0.99974698 | 2 |
| MG3-6 | 2 | TGGGTGAGTGAGTGTGTGCGTG | TGGGGTT | 99.65 | 16471 | 0.35856961 | 13 |
| MG3-6 | 3 | GAGGCCCTGGGCAGGTTGGTAT | CAAGGTT | 95.375 | 3372 | 1 | 0 |
| MG3-6 | 4 | AGGTTGGTATCAAGGTTACAAG | ACAGGTT | 92.95 | 1604 | 0.98004988 | 2 |
| MG3-6 | 5 | TTTAAATTATGAATCCATCTCT | AAAGGTT | 97.25 | 621 | 1 | 0 |
| MG3-6 | 6 | AGGCCTGTGTGTGTGTCTCCGT | TCGGGTT | 99.045 | 24302 | 0.98391079 | 2 |
| MG3-6 | 7 | aggaatctgcctaacaggaggt | GGGGGTT | 98.535 | 1608 | 1 | 0 |
| MG3-6 | 8 | tagtgttagtacagttttgctg | AAAGGTT | 58.66 | 175 | 1 | 0 |
| MG3-6 | 9 | attttgtatttgtgaagtctta | CAAGGTT | 94.65 | 211 | 1 | 0 |
| MG3-6 | 10 | gaagttggtggtgaggccctgg | GCAGGTT | 97.5 | 3744 | 0.52751068 | 1 |
|  |  |  |  |  |  |  |  |
| MG3-8 | 1 | CGGTGGCACTGCGGCTGGAGGT | GGGGGTT | 92.79 | 21324 | 1 | 0 |
| MG3-8 | 2 | TGGGTGAGTGAGTGTGTGCGTG | TGGGGTT | 97.73 | 16675 | 0.70932534 | 3 |
| MG3-8 | 3 | gaggccctgggcaggttggtat | CAAGGTT | 88.155 | 6322 | 1 | 0 |
| MG3-8 | 4 | aggttggtatcaaggttacaag | ACAGGTT | 43.145 | 1644 | 1 | 0 |
| MG3-8 | 5 | tttaaattatgaatccatctct | AAAGGTT | 48.83 | 337 | 1 | 0 |
| MG3-8 | 6 | AGGCCTGTGTGTGTGTCTCCGT | TCGGGTT | 92.58 | 7488 | 1 | 0 |
| MG3-8 | 7 | aggaatctgcctaacaggaggt | GGGGGTT | 91.86 | 1329 | 1 | 0 |
|  |  |  |  |  |  |  |  |
| SpCas9 | 1 | GGCACTGCGGCTGGAGGTGG | GGG | 96.19 | 26441 | 0.11682614 | 74 |
| SpCas9 | 2 | GGTGAGTGAGTGTGTGCGTG | TGG | 83.525 | 10094 | 0.34040024 | 25 |
| SpCas9 | 3 | GGCCCTGGGCAGGTTGGTATCA | AGG | 92.805 | 1277 | 0.85636222 | 7 |
| SpCas9 | 4 | GTTGGTATCAAGGTTACAAGAC | AGG | 86.53 | 491 | 1 | 0 |

**Supplementary Table 6: Amino acid boundaries for chimera design.**

| **Name** | **REC+NUC** | **PI** |
| --- | --- | --- |
| MG3-6+MG1-4 | MG3-6 (1-742) | MG1-4 (750-1025) |
| MG3-6+MG1-5 | MG3-6 (1-742) | MG1-5 (789-1077) |
| MG3-6+MG1-6 | MG3-6 (1-742) | MG1-6 (773-1059) |
| MG3-6+MG1-7 | MG3-6 (1-742) | MG1-7 (775-1061) |
| MG3-6+MG2-4 | MG3-6 (1-742) | MG2-4 (876-1201) |
| MG3-6+MG2-7 | MG3-6 (1-742) | MG2-7 (817-1080) |
| MG3-6+MG3-3 | MG3-6 (1-742) | MG3-3 (750-1132) |
| MG3-6+MG3-4 | MG3-6 (1-742) | MG3-4 (743-1134) |
| MG3-6+MG3-7 | MG3-6 (1-742) | MG3-7 (751-1131) |
| MG3-6+MG3-8 | MG3-6 (1-742) | MG3-8 (741-1132) |
| MG3-6+MG4-2 | MG3-6 (1-742) | MG4-2 (747-1043) |
| MG3-6+MG4-5 | MG3-6 (1-742) | MG4-5 (747-1055) |
| MG3-6+MG6-3 | MG3-6 (1-742) | MG6-3 (709-1027) |
| MG3-6+MG14-1 | MG3-6 (1-742) | MG14-1 (756-1003) |
| MG3-6+MG15-1 | MG3-6 (1-742) | MG15-1 (729-1082) |
| MG3-6+MG16-1 | MG3-6 (1-742) | MG16-1 (787-1154) |
| MG3-6+MG16-2 | MG3-6 (1-742) | MG16-2 (796-1227) |
| MG3-6+MG18-1 | MG3-6 (1-742) | MG18-1 (997-1348) |
| MG3-6+MG21-1 | MG3-6 (1-742) | MG21-1 (740-1098) |
| MG3-6+MG22-1 | MG3-6 (1-742) | MG22-1 (1092-1521) |
| MG3-6+MG23-1 | MG3-6 (1-742) | MG23-1 (1008-1377) |
| MG3-6+SaCas9 | MG3-6 (1-742) | SaCas9 (706-1053) |
| MG3-6+SpCas9 | MG3-6 (1-742) | SpCas9 (988-1368) |
| MG3-6+MG15-1 (WP) | MG3-6 (1-840) | MG15-1 (729-1082) |
| MG3-6+MG15-1 (P) | MG3-6 (1-742) | MG15-1 (931-1082) |


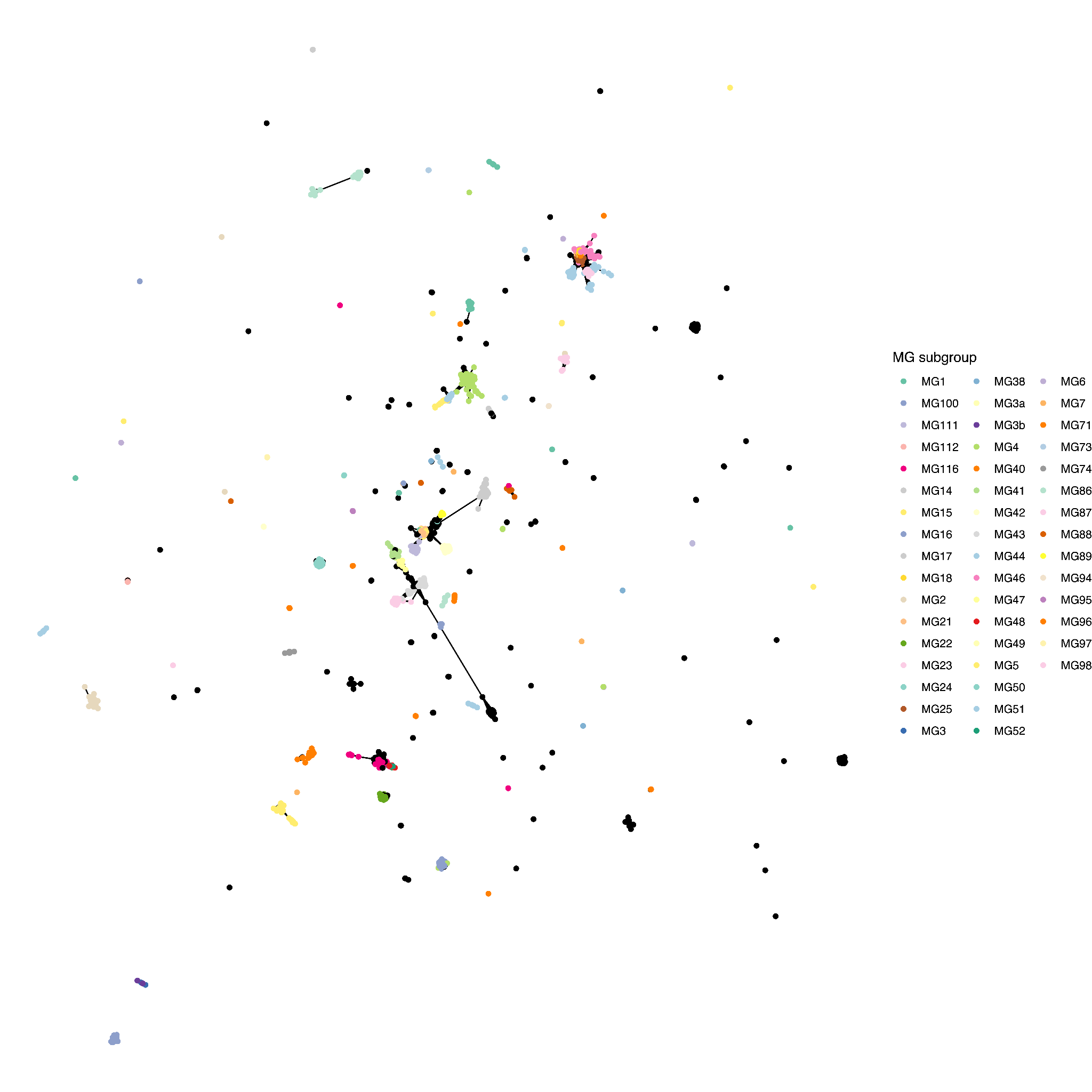


**Supplementary Figure S1:** Sequence similarity network of type II CRISPR effectors. Edges indicate a pairwise identity of > 40% over 50% of the query sequence. Nodes are colored according to the manually defined MG subgroup. Black nodes are reference sequences.

**
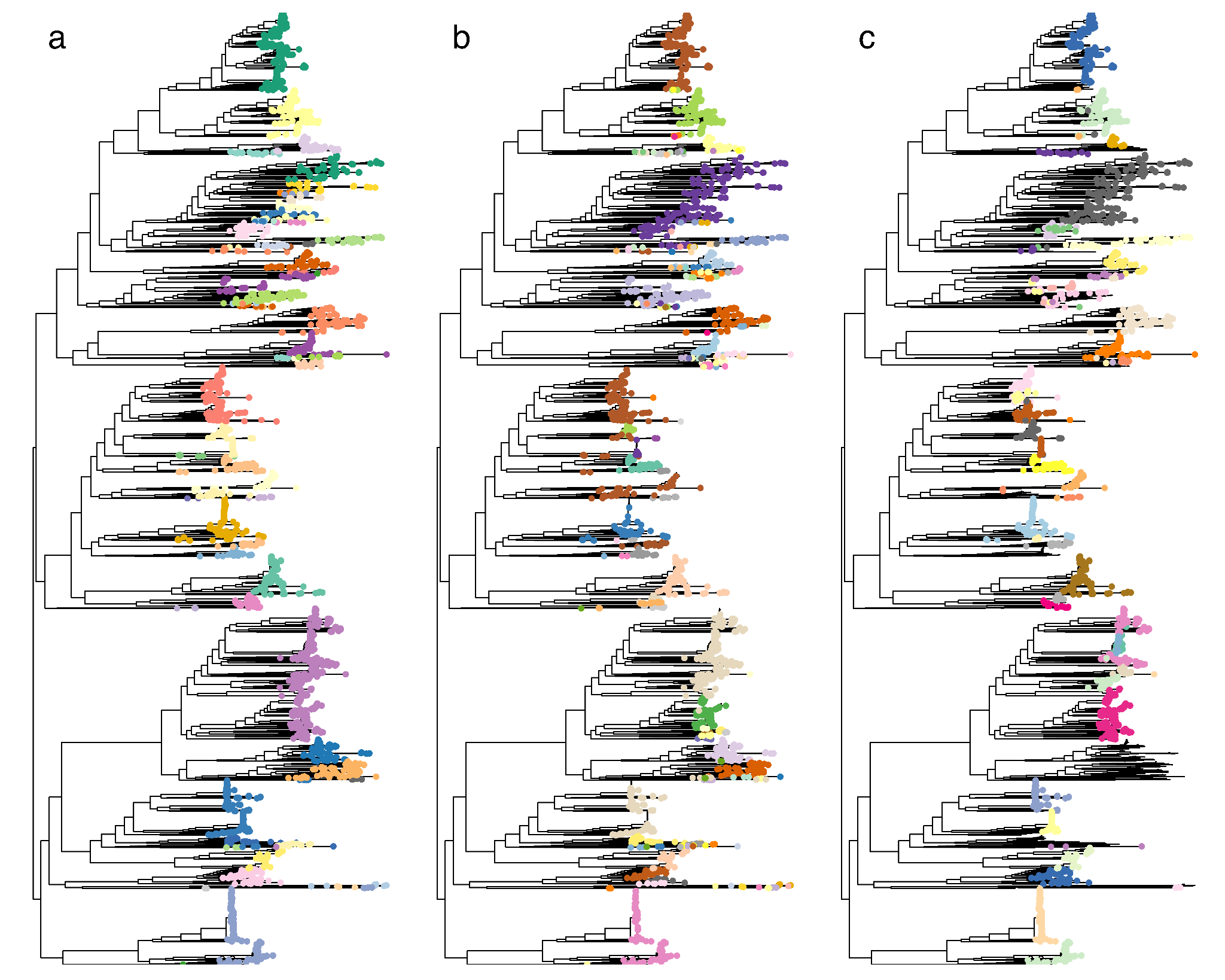
**

**Supplementary Figure S2:** Phylogenetic tree of type II CRISPR effectors from Figure 1a colored according to the a) TreeCluster clusters, b) sequence similarity network (SNN) clusters, and c) manually defined MG subgroups. Clustering methods are in agreement with adjusted Rand indices of 0.722, 0.724, and 0.853 when comparing SSN clusters to MG subgroups, TreeCluster clusters to MG subgroups, and TreeCluster clusters to SSN clusters, respectively. Tips lacking a color in c) correspond to reference sequences.

**
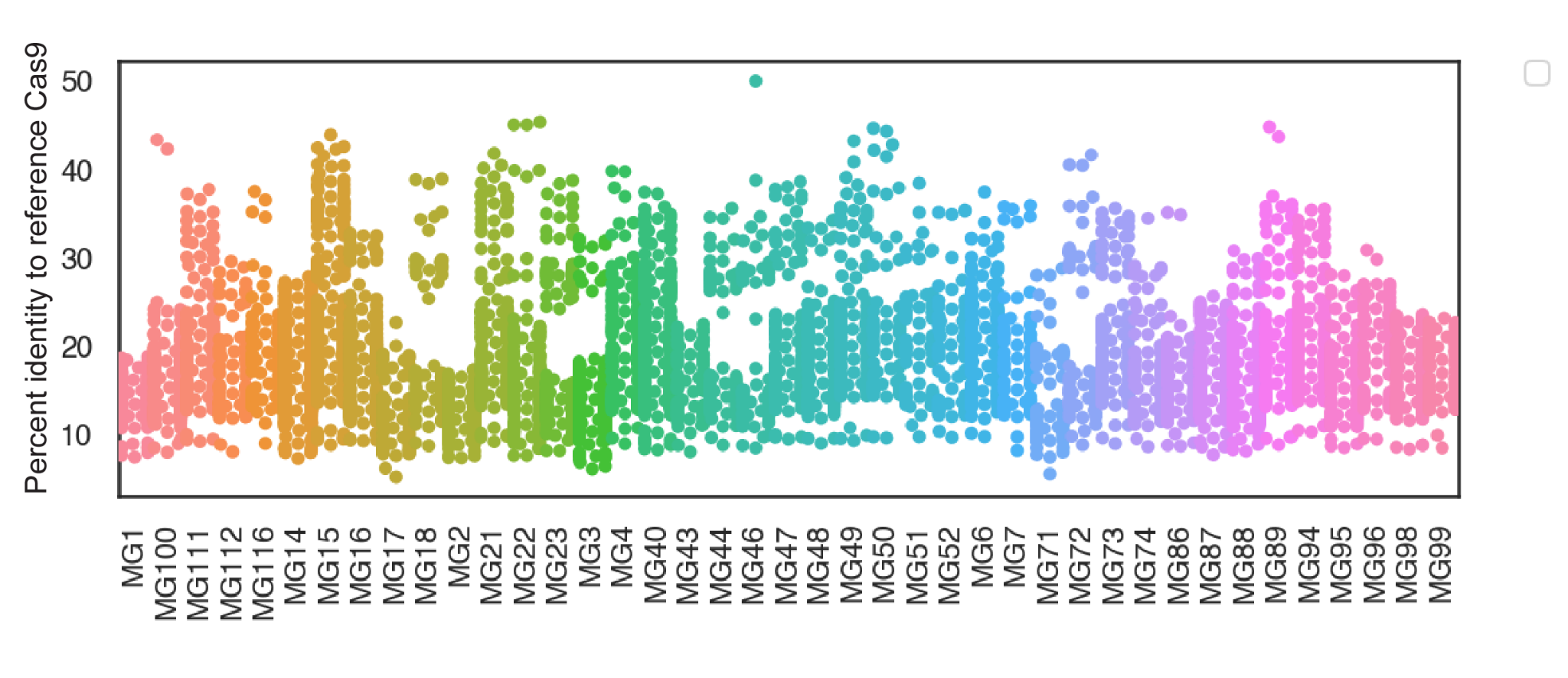
**

**Supplementary Figure S3: Percent identity distribution of novel MG Type II nucleases vs. reference Cas9.**

Percent identity of each of >3,400 unique novel nucleases (colored dots) vs. one of 84 reference sequences from a set of common active Cas9 nucleases and those reported in Gasiunas et al, 2020. Percent identity was determined from an identity matrix calculated with pseqsid (https://github.com/amaurypm/pseqsid) from the multiple sequence alignment used to infer the phylogenetic tree in Figure 1.


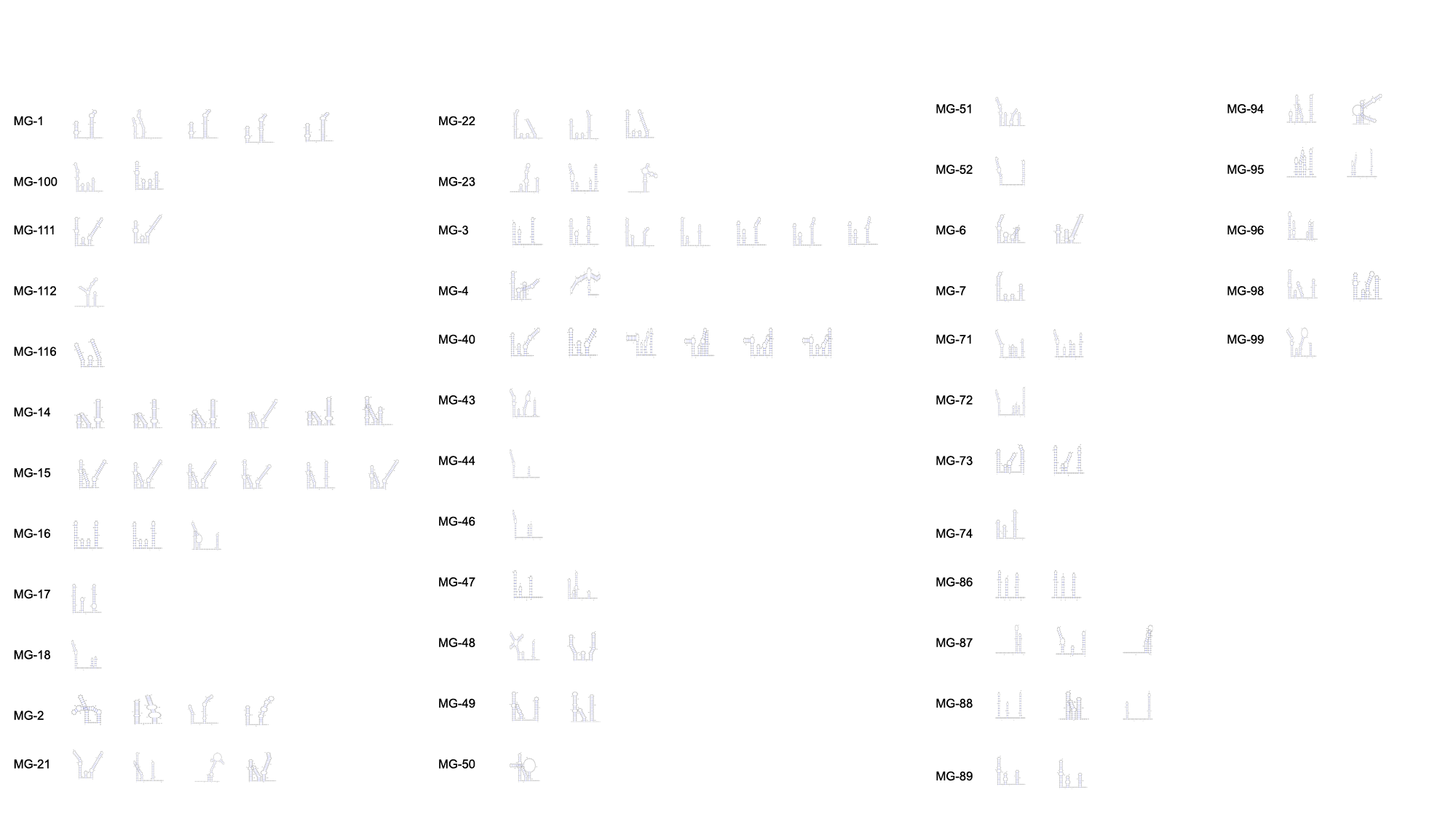


**Supplementary Figure S4: Tracr sequences of the active nucleases.**

After determining the guide sequence for each of the effectors, secondary structure models were constructed for each guide sequence using RNAalifold 2.5.0 (-p --aln-stk). Thereafter, VARNA (VARNAv3-93) was utilized for visualizing the RNA secondary structure, that was predicted by RNAalifold, of each guide sequence.

**
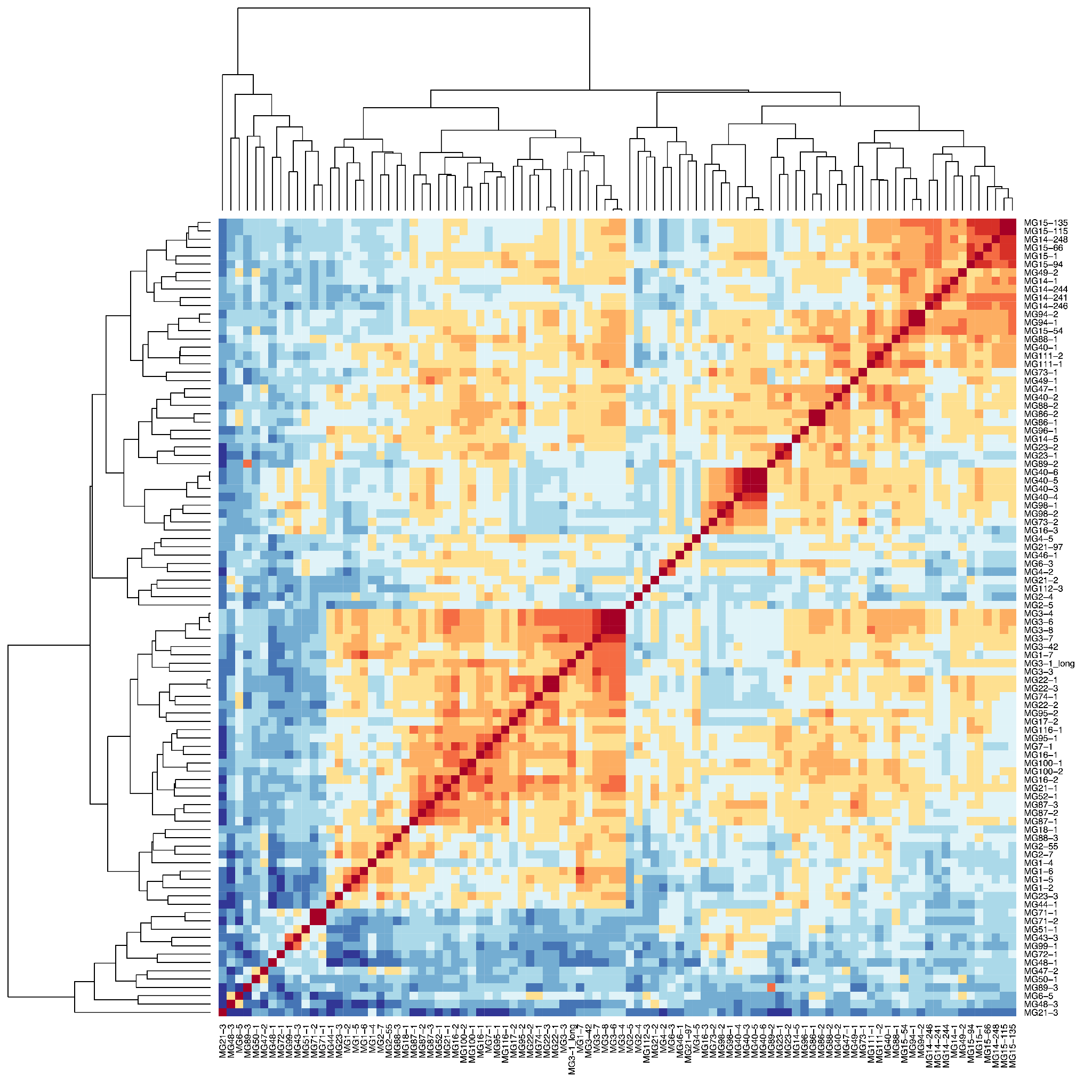
**

**Supplementary Figure S5:** Heatmap of the pairwise similarities between thermodynamic TracrRNA secondary structures. Dark red indicates high similarity and dark blue indicates low similarity.


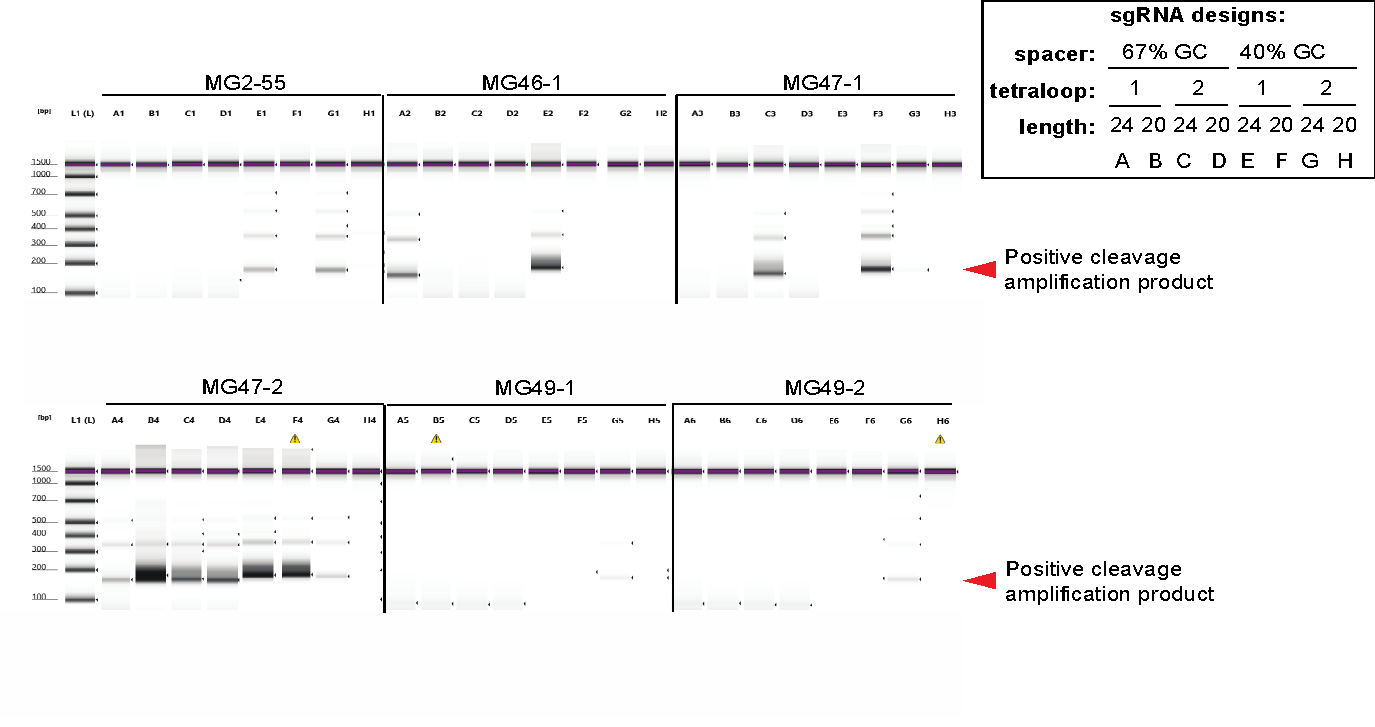


**Supplementary Figure S6: Enrichment screening data for six nucleases showing activity with different guide designs.**

The enrichment signal is a PCR band detectable at ~180 bp. Here we see the various activities for eight different guide designs. The sgRNAs use two different spacers, two different spacer lengths, and two different positions where the tetraloop is inserted between the repeat and tracrRNA. For candidate MG2-55, it has both a spacer preference (spacer 2) and requires a 24 nt spacer, but is flexible with regards to tetraloop position (guides E or G). For MG46-1, it is only successful with a single tetraloop position and spacer length, but with either spacer (guides A or E). For MG47-2, the guide design is much more flexible for detectable activity but guide B was used as the preferred guide design.


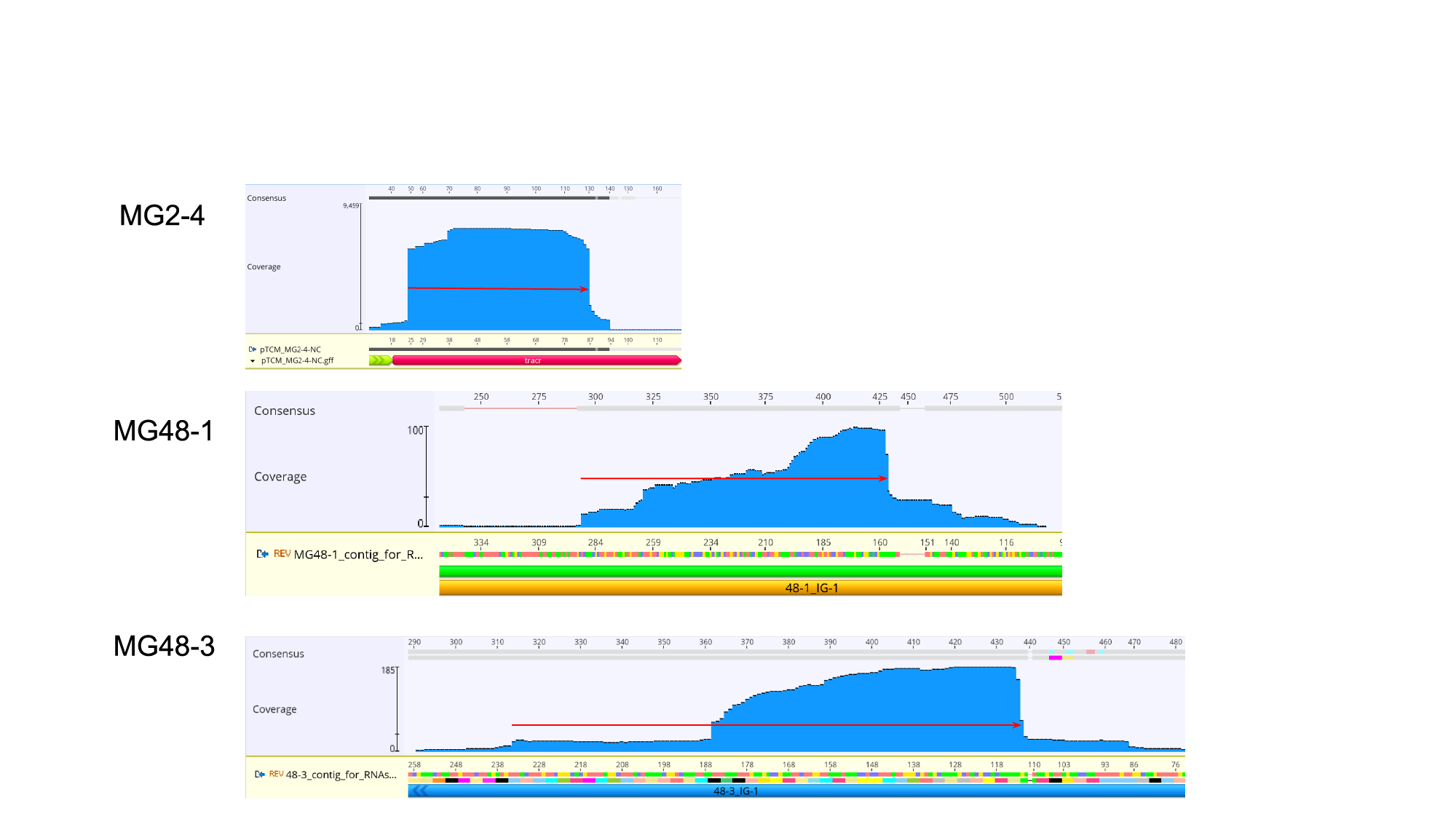


**Supplementary Figure S7: RNAseq reads for tracr identification.**

RNAseq for MG2-4 shows that the initially predicted tracr (in pink) was too long. The reads are shown in blue and the active tracr region is denoted by the red arrow. For 48-1 and 48-3 the intergenic region upstream of the nuclease did contain the correct tracr, shown by the red arrow. All reads are shown in the forward direction.


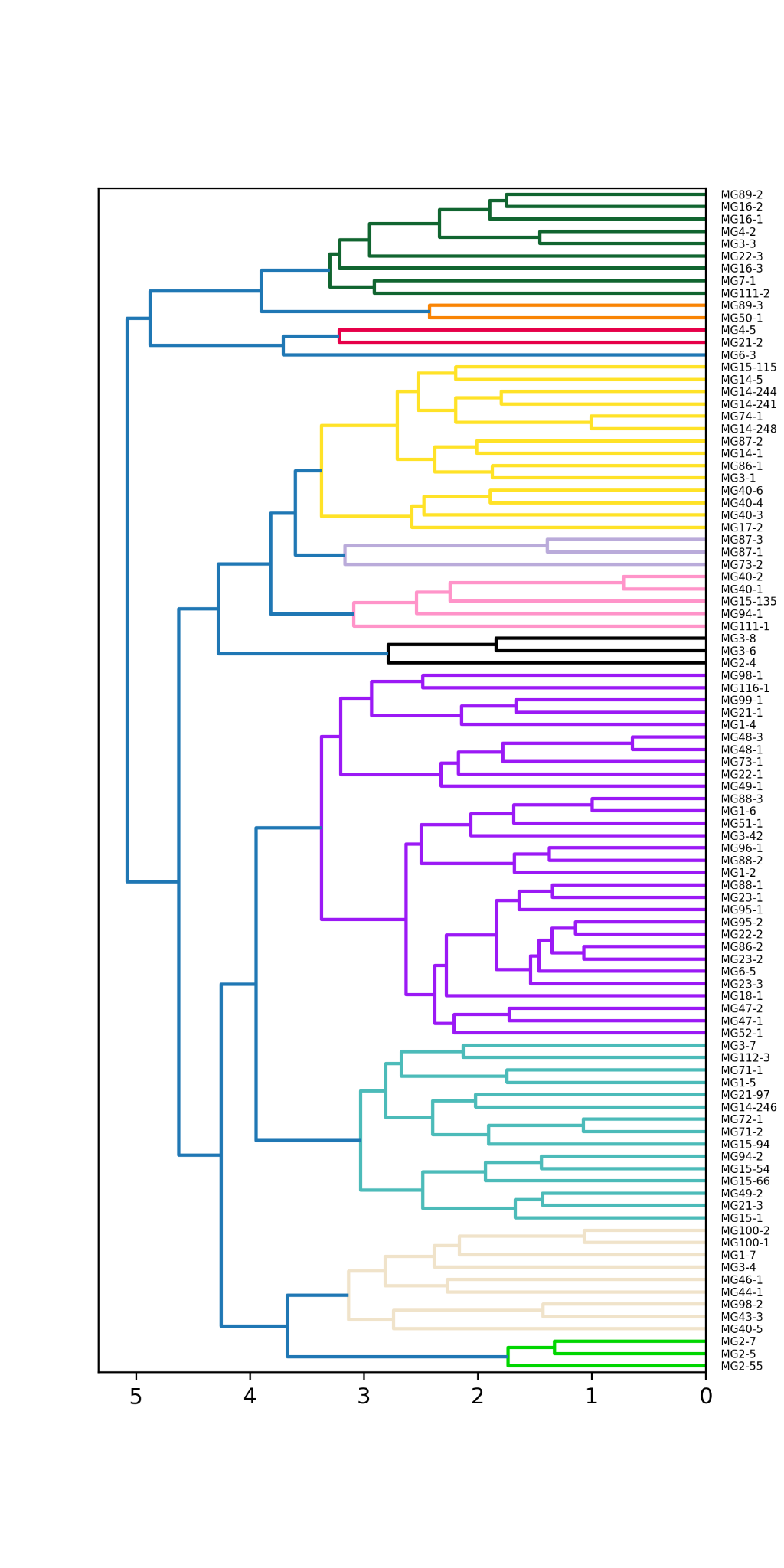


**Supplementary Figure S8: hierarchical clustering of PAM motifs.**

Clustering was estimated from the Euclidean distance of nucleotide frequency from the in vitro experiments and reveals 12 distinct groups of PAMs.


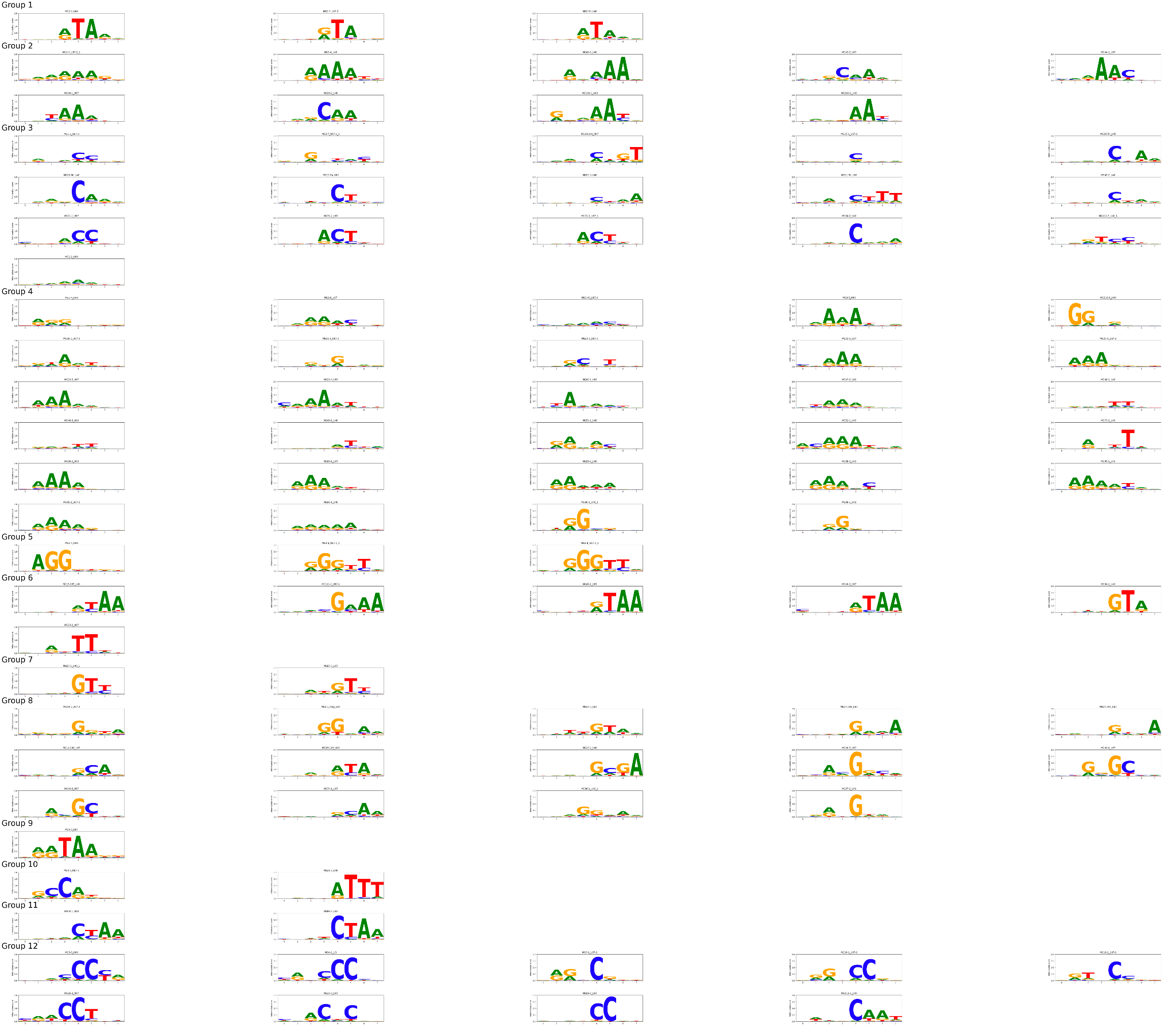


**Supplementary Figure S9: The PAMs determined by the *in vitro* screen for novel nucleases.**

PAM y-axis is the bit score of information content. The PAMs are determined for a maximum of 8 adjacent bases. PAMs are arranged according to the hierarchical clusters in Figure S8.


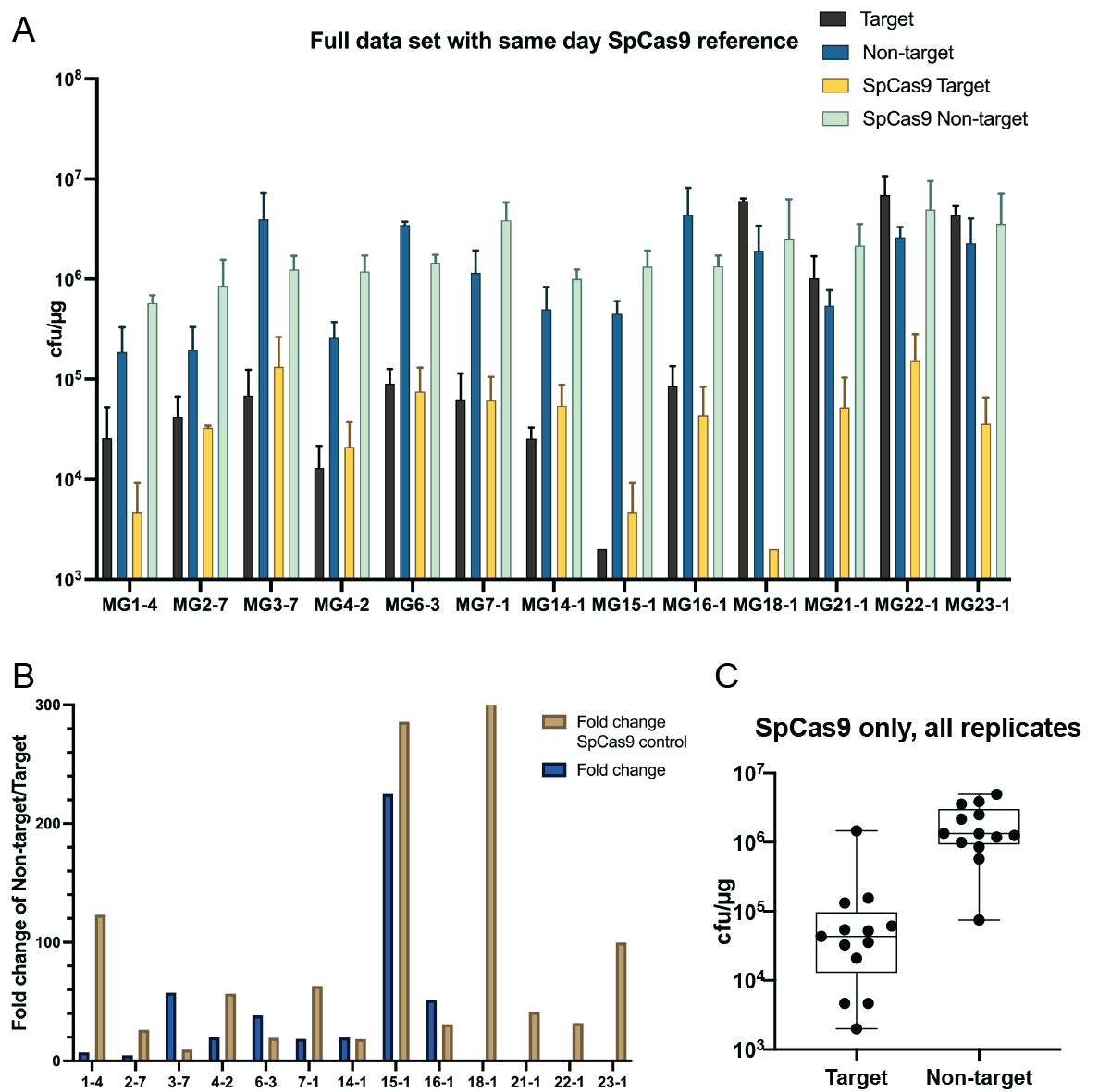


**Supplementary Figure S10: *E. coli* activity assay data with same day controls.**

**(A)** The raw data from genomic targeting *E. coli* experiments (N=3, same day replicates). **(B)** The data from **A** shown as a ratio of non-target to target growth repression. **(C)** SpCas9 from all replicates. Growth and transformation are variable, requiring normalization controls for cross-day comparisons. Each data point is the average of three, one day replicates.


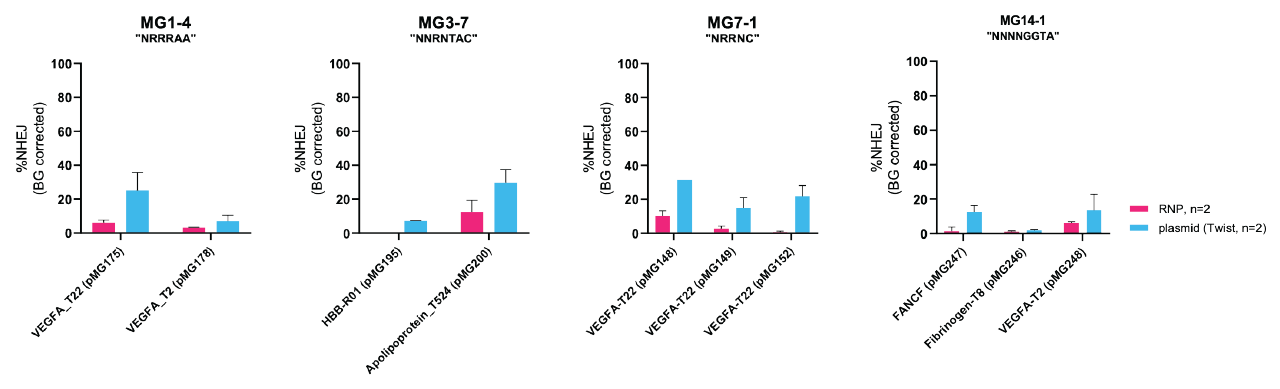


**Supplementary Figure S11: RNP vs plasmid editing data for four additional nucleases.**

Unlike the MG3 proteins, these proteins did not improve their editing activity when purified.


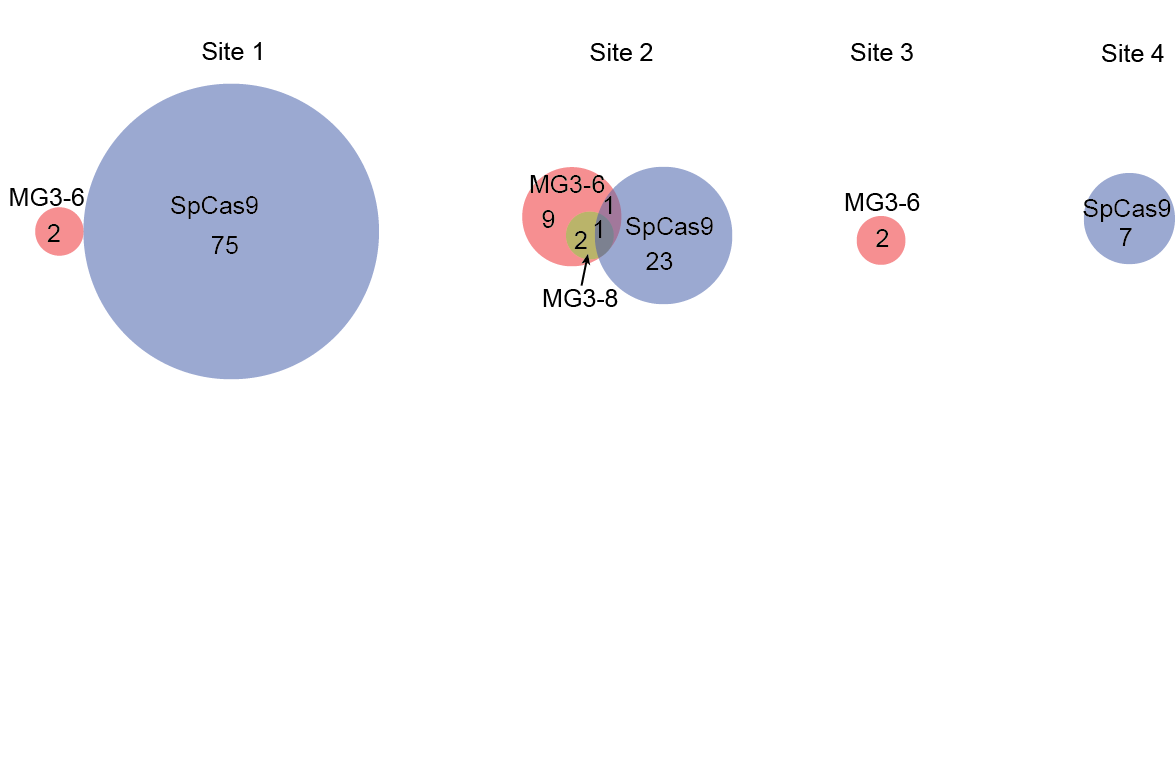


**Supplementary Figure S12: Number of off-target sites identified via oligo-capture NGS data.**

Some of the off-target sites are the same, however most are unique to MG3-6 or SpCas9. MG3-8 overlaps entirely with MG3-6 when off-targets are detected.


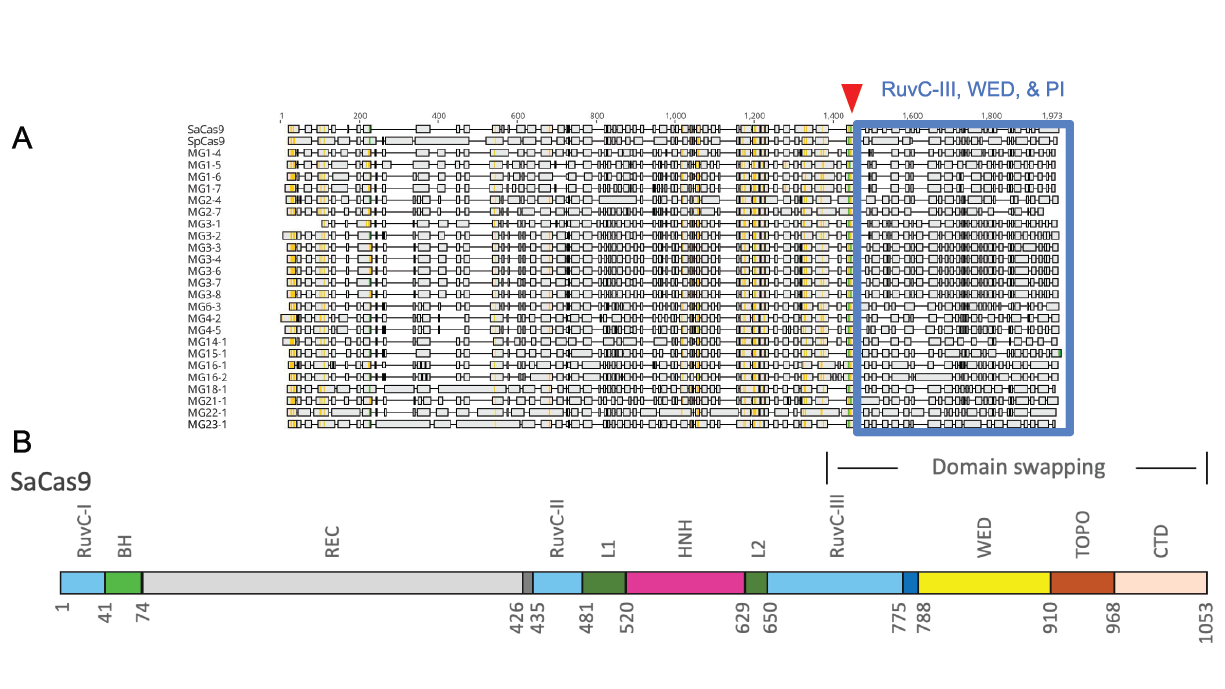
**Supplementary Figure S13: Chimeric protein design.**

**(A)** The structural alignment identifies a conserved two residue patch that can be used for swapping designs (red arrow). **(B)** The C-terminal region that is removed and swapped is shown for the gene diagram of SaCas9.
